# Mechanophysical Synthesis of Core/Shell Hybrid Supraparticles

**DOI:** 10.1002/adma.202502718

**Published:** 2025-04-24

**Authors:** Jeonguk Hwang, Seong Hwan Lee, Jinsu Kim, Geonho Lee, Jinwoo Park, Yunseok Choi, Jinhoon Lee, Jin Hong Lee, Jae Ryung Choi, Cheol‐Min Yang, Il Jin Kim, Bo‐In Park, Shu Yang, Seung‐Yeol Jeon, Dong Woog Lee, Seunggun Yu

**Affiliations:** ^1^ School of Energy and Chemical Engineering Ulsan National Institute of Science and Technology (UNIST) Ulsan 44919 Republic of Korea; ^2^ Insulation Materials Research Center Korea Electrotechnology Research Institute (KERI) Changwon 51543 Republic of Korea; ^3^ Institute of Advanced Composite Materials Korea Institute of Science and Technology (KIST) Jeollabuk‐do 55324 Republic of Korea; ^4^ Composite Research Division Korea Institute of Materials Science (KIMS) Changwon 51508 Republic of Korea; ^5^ Department of Mechanical Engineering and Materials Science Washington University in Saint Louis Saint Louis 63130 USA; ^6^ Department of Organic Materials Science and Engineering Pusan National University Busan 46241 Republic of Korea; ^7^ Fusion Materials Research Group Korea Institute of Materials Convergence Technology (KIMCO) Busan 47154 Republic of Korea; ^8^ Department of Semiconductor Science and Technology Jeonbuk National University Jeollabuk‐do 54896 Republic of Korea; ^9^ Department of Materials Science and Engineering University of Pennsylvania Philadelphia 19104 USA; ^10^ Department of JBNU‐KIST Industry‐Academia Convergence Research Jeonbuk National University Jeollabuk‐do 54896 Republic of Korea; ^11^ Electro‐functional Materials Engineering University of Science and Technology (UST) Changwon 51543 Republic of Korea

**Keywords:** assembly, catalyst, collision, mechanophysical synthesis, supraparticles

## Abstract

Surface modification of polymer microparticles (MPs) is often essential to impart functionalities beyond their inherent properties. However, decorating these surfaces typically requires complex, multi‐step wet chemistry processes to direct assembly and bonding between surfaces, which are not only challenging to control and scale up but also pose significant environmental concerns. Inspired by asteroid impact events, assembly of core/shell hybrid supraparticles (HSPs) is demonstrated via collision‐driven, one‐step dry mixing of inorganic nanoparticles (NPs) and polymer MPs with a significant contrast in elastic moduli— a process termed “mechanophysical synthesis.” Through the interplay of interfacial energy and collision energy, NPs are stably embedded onto the MP surface. The degree of surface coverage depends on mixing velocity and duration, aligning with results from particle collision simulations. HSPs can be created from a diverse combination of MPs and NPs, regardless of their shapes or chemistry. Furthermore, different types of functional NPs—such as magnetic, photocatalytic, and ion‐adsorptive—can be simultaneously introduced onto the MPs. The resulting HSPs can not only remove toxic water pollutants, but also be easily recovered and reused. The mechanophysical synthesis approach opens a new direction for sustainable and versatile self‐assembly of heterogeneous MPs, minimizing the use of excessive chemicals and solvents.

## Graphical abstract

Collision‐driven mechanophysical synthesis enables rapid, chemical‐free assembly of core/shell hybrid supraparticles (HSPs) by embedding inorganic nanomaterials onto polymer microparticles. Governed by collision and interfacial energies, this scalable, solvent‐free method accommodates diverse material combinations, allowing multifunctional HSPs for pollutant removal with easy recovery and reuse–offering a sustainable alternative to conventional surface modification techniques, which often require excessive chemicals and multi‐step processes.

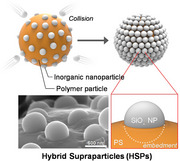

## 1 Introduction

**Figure 1:**
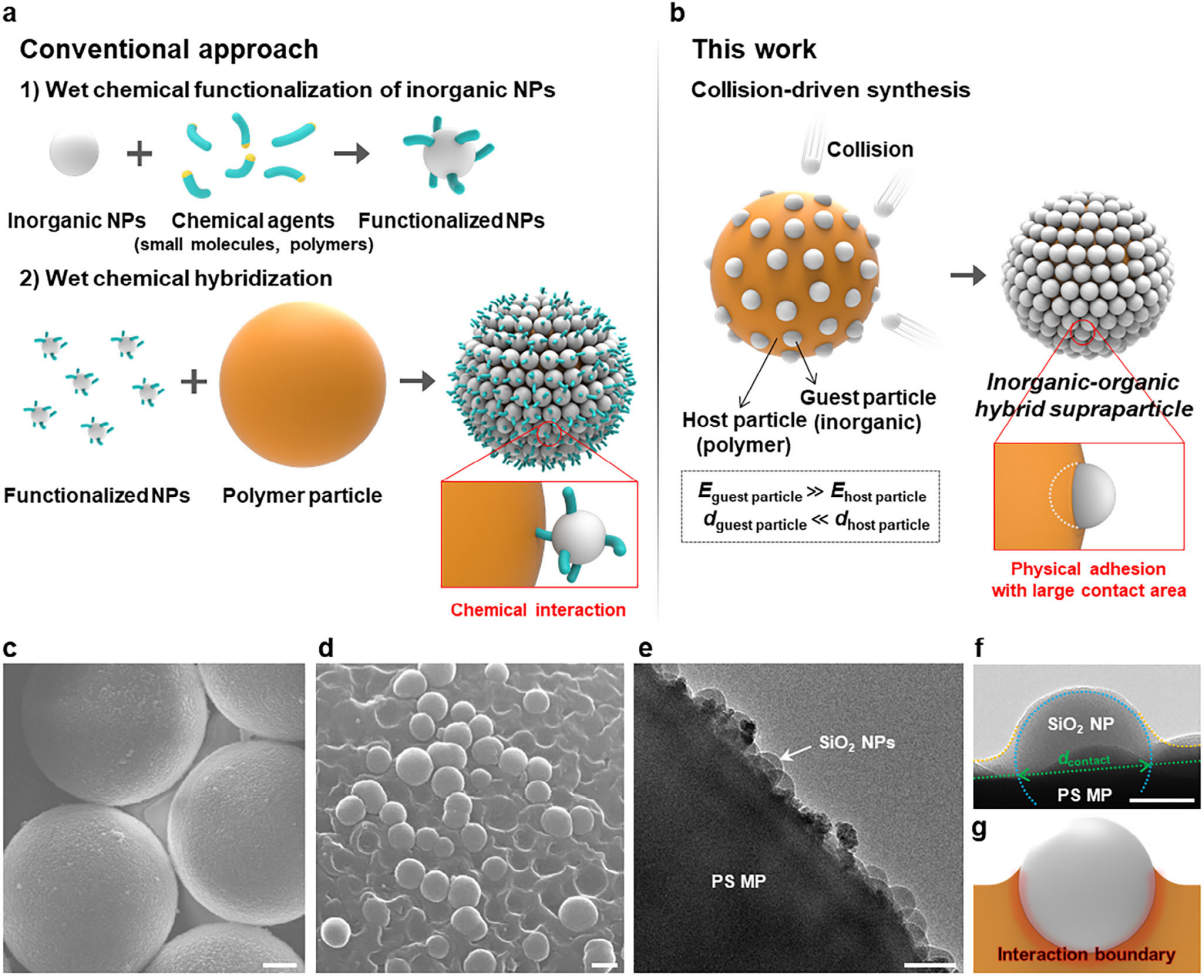
Synthesis of organic–inorganic HSPs through mechanophysical assembly. a,b) Schematic illustration of the conventional approach based on multi‐step wet chemistry (a) and one‐step collision‐driven assembly (b). The HSPs are obtained when the modulus (*E*) of the guest inorganic NPs is higher and the diameter (*d*) is smaller than those of the host polymer particle. c,d) SEM images of SiO_2_/PS HSPs with a diameter of 10 µm (c) and SiO_2_ NPs adhered onto the PS MP (d), leaving craters after the NPs are removed. e,f) Cross‐sectional TEM images of the SiO_2_/PS HSP (e) and its magnification (f). The blue, yellow, and green dotted lines indicate the boundaries of SiO_2_ NP, PS ridge formed along the surface SiO_2_ NP, and PS MP, respectively. In (f), the contact diameter (*d*_contact_) is indicated by the arrow. g) Scheme depicting the TEM image in (f). The scale bars denote 2 µm for (c), 100 nm for (d), 100 nm for (e), and 50 nm (f).

Polymer microparticles (MPs) with surfaces decorated with diverse nanomaterials to impart additional functionalities that are not inherently present are of significant interest for numerous applications including photonics,^[^ ^1^, ^2^, ^3^ ^]^ drug delivery,^[^ ^4^, ^5^, ^6^ ^]^ sensors,^[^ ^7^, ^8^ ^]^ catalysts^[^ ^9^, ^10^, ^11^ ^]^ and wetting.^[^ ^12^, ^13^ ^]^ For example, core/shell,^[^ ^14^, ^15^, ^16^ ^]^ patchy,^[^ ^17^, ^18^, ^19^, ^20^ ^]^ or supraparticles^[^ ^21^, ^22^, ^23^ ^]^ with small molecules,^[^ ^24^, ^25^ ^]^ macromolecules,^[^ ^26^, ^27^, ^28^ ^]^ and inorganic nanoparticles (NPs)^[^ ^11^, ^14^, ^29^, ^30^, ^31^, ^32^ ^]^ have been synthesized via “wet chemistry.” This process involves multiple steps, including the dispersion and hybridization of the particles, followed by washing, isolation of the NPs, and removal of unreacted chemicals (**Figure** **1**a).^[^ ^33^, ^34^ ^]^ For example, small molecules or polymers are dispersed in a solvent and mixed with NPs followed by covalent bonding of small molecules or polymers on NPs. Subsequently, these chemically modified NPs are mixed with the polymer MPs, wherein the chemical moieties on the NP's surface interact with the surface groups on polymer MPs covalently or non‐covalently to form polymer MP‐core/inorganic NP‐shell hybrid particles. Depending on the application, these hybrid particles may undergo additional processes such as solvent exchange, drying, and washing, which raise concerns about scalability and environmental impact. Therefore, a novel approach that minimizes the processing steps and energy consumption without sacrificing performance is highly desired.

Here, we draw inspiration from asteroid impact events, such as those involving meteors and comets, to synthesize hybrid supraparticles (HSPs) without the use of solvents or other chemicals. When asteroids collide with planetary bodies like the Earth, they often leave behind traces of depressions known as craters, such as *Barringer Crater* and *Chicxulub Crater* (Figure S1, Supporting Information).^[^ ^35^, ^36^ ^]^ Despite asteroids typically being less than 0.1% the size of Earth and composed of hard rock up to ≈100 km thick, craters are formed when these high‐velocity asteroids collide with the surface, releasing immense energy that excavates the impacted area.^[^ ^37^ ^]^

By inducing collisions through powder mixing, we demonstrate that the NPs can be loaded onto the polymer MPs via surface deformation, mimicking the impact events of asteroids. Therefore, we term this process “mechanophysical synthesis.” We present the fabrication of multivalent HSPs using various NPs with diverse dimensions and properties as building blocks. Furthermore, we highlight their multifunctionality featuring magnetic, photocatalytic, and adsorptive capabilities integrated within a single particle.

## 2 Synthesis of Organic–Inorganic HSPs

The concept of mechanophysical synthesis of HSP, involving the mechanical collision of the NPs and polymer MPs as guest and host particles, respectively, is illustrated in Figure 1b. When the hard NPs with a larger elastic modulus (*E*) and a smaller diameter (*d*) compared to the polymer MPs, are vigorously mixed, numerous contacts are generated through random collisions. As a result, the surface of the softer polymer MPs becomes deformed, allowing the collided inorganic NPs to adhere with large contact areas and indentations, facilitated by the large differences in *E*.^[^ ^39^ ^]^ Thus, HSPs can be simply fabricated by dry mixing the guest and host particles even at room temperature, as long as they satisfy specific modulus and size requirements. The number of synthesis steps (*N_s_*) of the HSP is just 1, whereas the conventional wet chemistry route involves at least four steps, including dispersion, surface functionalization, washing of NPs in a solution, and chemical reactions with the polymer host particles.

To investigate the dry mixing process, we use SiO_2_ NPs (*E*_SiO2_: ≈74.0 GPa) with a diameter of 100 nm as model guest particles and polystyrene (PS) MPs (*E*_PS_: ≈3.4 GPa) with a diameter of 10 µm as model host particles. These were mixed in a vial at a specific ratio using a magnetic stirrer set to 700 revolutions per minute (RPM) for 30 min at room temperature (Figure S2, Supporting Information). After mixing, the entire surface of the PS MPs is coated with SiO_2_ NPs (Figure 1c), which was confirmed by low‐magnification scanning electron microscopy (SEM) and differential interference contrast (DIC) images showing fine distribution (Figure S3, Supporting Information). Following intensive sonication for 30 min, approximately half of the NPs are detached, leaving behind the traces of the “craters” on polymer MPs, indicating where the NPs were previously attached (Figure 1d). Additionally, some SiO_2_ NPs remain individually embedded on the surface of the soft PS MPs. A side view clearly reveals the formation of a meniscus of the PS particle along the periphery of the embedded SiO_2_ NPs (Figure 1e,f; Figure S4, Supporting Information). This collision process between the SiO_2_ and PS enhances the physical interactions by increasing the contact area (Figure 1g).

Beyond PS, SiO_2_ NPs can also be assembled on the surfaces of various polymer particles, including polyphenylene sulfide, polyamide 6, polymethyl methacrylate, and polyethylene through the same collision‐based methods (Figure S5, Supporting Information).

Based on the simple yet robust nature of this approach, a large quantity of the HSPs was successfully synthesized using our custom‐designed batch‐type apparatus (Figure S6, Supporting Information). This demonstrated that the process does not rely on specialized equipment and could be readily scaled up using commercially available mixing units.

## 3 Adhesion Principle Through Mechanophysical Synthesis

**Figure 2:**
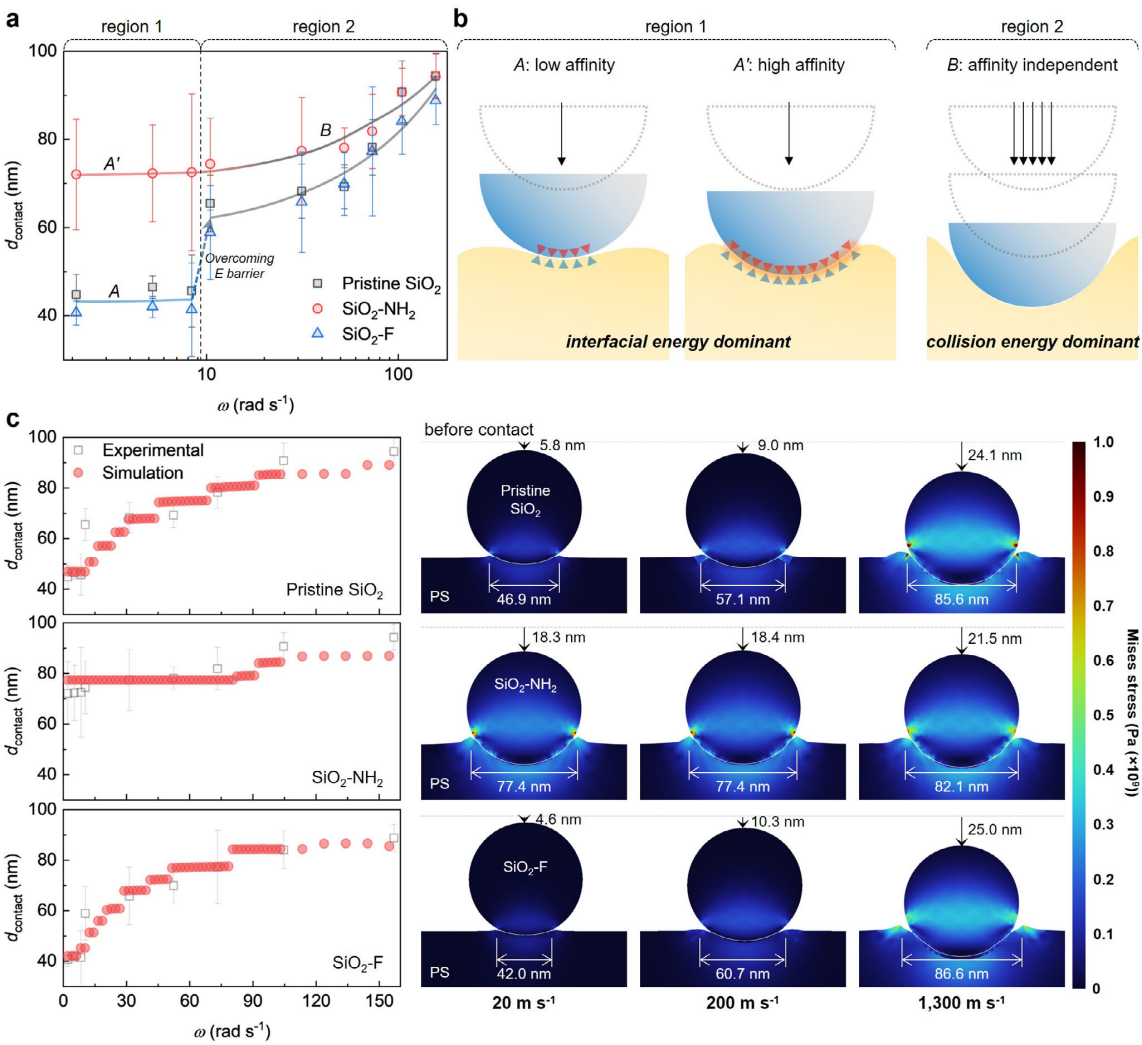
The adhesion principle between the SiO_2_ NPs and PS MPs. a) *d*_contact_ of the pristine SiO_2_ NPs, SiO_2_‐NH_2_ NPs, and SiO_2_‐F NPs collided onto the PS MP as a function of the angular frequency (*ω*). b) Schematic illustrations of the adhesion processes of the SiO_2_ NPs onto the PS surface. In region 1, the interfacial energy is dominant to determine the affinity of the SiO_2_ NPs. In region 2, the collision energy is dominant to determine the embedment depth. c) Simulated contact diameter (left) and contact geometry and corresponding von Mises stress distribution at different collision speeds (20, 200, and 1300 m s^−1^) (right).

To gain deeper insights into the physical mechanism, we measured the diameter of the craters (contact diameter, *d*_contact_) to analyze the surface deformation and adhesion behavior under varying mixing velocity. In **Figure** **2**a, we analyze the impaction processes of SiO_2_ NPs by examining the changes in *d*_contact_ as a function of angular frequency (*ω*) at a fixed collision time of 10 min. At the lowest *ω* of 2.1 rad s^−1^, the collision of the NPs (*d* = 100 nm) formed craters with a diameter of ≈45 nm (black square). In the low *ω* regime (<9 rad s^−1^, region 1), *d*_contact_ shows minimal variation with increasing *ω*. We also investigate the contact behaviors of SiO_2_ NPs with varying surface energies (per unit area) by functionalizing the pristine SiO_2_ NPs with (3‐aminopropyl)triethoxysilane (APTES) and fluorotrimethylsilane (FTMS), respectively (Figure S7, Supporting Information). The surface energies of the SiO_2_ NPs were estimated by treating Si wafers with each modifier and performing contact angle measurements, followed by calculations based on the Owens–Wendt method.^[^ ^38^ ^]^ The resulting surface energies for the pristine Si wafer (SiO_2_), FTMS‐treated Si wafer (SiO_2_‐F), and APTES‐treated Si wafer (SiO_2_‐NH_2_) were determined to be 55.45 ± 2.36, 28.69 ± 2.23, and 62.95 ± 1.40 mJ m⁻^2^, respectively (Table S1, Supporting Information). Furthermore, the interfacial energies between PS and each SiO_2_ were determined by using the Good‐Girifalco equation (Note S1. Methods, Supporting Information). Compared to the PS/SiO_2_ interface (interfacial energy, γ = 2.61 ± 0.23 mJ m^−2^), the PS/SiO_2_‐F interface, with lower interfacial energy (γ = 0.23 ± 0.09 mJ m^−2^) than the PS/SiO_2_ interface, exhibit a smaller *d*_contact_ of ≈40 nm (blue triangle), which remains relatively constant in the low *ω* regime. In contrast, PS/SiO_2_‐NH_2_ interfaces, with higher interfacial energy (*γ* = 4.42 ± 0.17 mJ m^−2^) than the PS/SiO_2_ interface, exhibit a larger *d*_contact_ of ≈72 nm (red circle), attributed to their higher affinity and the interfacial energy balance.

In the low *ω* regime, the low collision energy results in small *d*_contact_ and limited contact areas, where the interfacial energy balance among the NPs (solid 1), the MPs (solid 2), and the air primarily governs their interactions.^[^ ^39^ ^]^ The surface energy of SiO_2_ NPs (specifically at the SiO_2_/air interface) predominantly influences the interfacial energy balance among the three phases (PS/SiO_2_/air). Due to its higher surface energy, the SiO_2_‐NH_2_ NP tends to minimize the SiO_2_/air interfacial area by penetrating more deeply into the PS core. Therefore, pristine SiO_2_ and SiO_2_‐F exhibit a lower work of adhesion with PS compared with SiO_2_‐NH_2_, resulting in smaller contact areas between these NPs and the PS MPs (Figure 2b).

In the high *ω* regime (>9 rad s^−1^, region 2), interestingly, the *d*_contact_ of pristine SiO_2_ and SiO_2_‐F, which have a lower affinity with PS compared to SiO_2_‐NH_2_, sharply increases to ≈65 and 60 nm, respectively, and continues to increase with increasing *ω*. This behavior suggests the presence of a distinct energy barrier between regions 1 and 2, which is overcome when sufficient collision energy is provided, resulting in a collision‐dependent impact process for the NPs. In contrast, the collision between SiO_2_‐NH_2_ NPs and PS MPs exhibits a smooth transition in *d*_contact_ from region 1 to 2. Therefore, in the high *ω* regime, collision energy becomes the dominant factor driving the formation of HSP. Additionally, the *d*_contact_ was analyzed by calculating the number of rotations (*N*_rotation_) as the product of RPM and time, further supporting that *d*_contact_ exhibits two distinct regions during HSP formation, where region 1 was *N*_rotation_‐independent and dominated by surface energy, while region 2 showed an increase in *d*_contact_ with higher *N*_rotation_. The difference between the two regions was statistically significant (*P* = 4.37 × 10^−23^) (Figure S8, Supporting Information).

This embedding behavior observed in our system clearly differs from ball milling or sandblasting, which rely on high‐energy input for material synthesis or polishing, typically leading to uncontrolled fusion or surface abrasion rather than localized and stable integration of NPs onto polymer MPs as seen in HSPs (Table S2, Supporting Information).

The effect of surface affinity on mechanophysical synthesis is further clarified through particle collision simulations that incorporate adhesion. The simulation assumed that *ω* is proportional to the impact velocity of the guest particles, which collide vertically with a fixed host particle (Figures S9–17 and Tables S3 and S4, Supporting Information). According to the simulations, the kinetic energy of the guest particle is dissipated through the viscoelastic deformation of the host particle, while the remaining energy is converted into elastic strain, contact, and adhesion energies within the host, leading to the embedding of the guest particles (Figure S18, Supporting Information).

The embedment depth (i.e., *d*_contact_) is determined by the difference between the repulsion force generated by the collision and the adhesion formed between the particles. When adhesion is sufficiently strong, the embedment depth remains constant even as the collision speed increases, because the repulsion force is offset by the adhesion (Figure S19, Supporting Information). The higher surface affinity between two particles enhanced adhesion, making the *d*_contact_ less sensitive to impact velocity, even at higher speeds (left in Figure 2c). For example, at a low collision speed of 20 m s^−1^, an increase in surface affinity (in the order of SiO_2_‐NH_2_, pristine SiO_2_, and SiO_2_‐F) results in larger *d*_contact_ and more deeply embedded guest particles. SiO_2_‐NH_2_ having the highest surface affinity, showed minimal changes in *d*_contact_ as collision speed increases. In contrast, for pristine SiO_2_ and SiO_2_‐F, the *d*_contact_ increased significantly with higher collision speeds (right in Figure 2c). These simulation results are consistent with the experimental data presented in Figure 2a.

It is noteworthy that the adhesion between PS MPs and SiO_2_ NPs also demonstrated not only considerable thermal stability at elevated temperatures (80 °C) but also chemical stability under various solvent environments (Figure S20, Supporting Information). While partial or complete detachment of NPs was observed under harsh acidic or basic conditions, the SiO_2_ NPs shell remained stably attached in neutral and mildly polar solvents. These results suggest that the interfacial bonding in our system is sufficiently robust to withstand most practical conditions, potentially offering chemical durability.

## 4 Assembly Behaviors of Self‐Limiting HSPs

**Figure 3:**
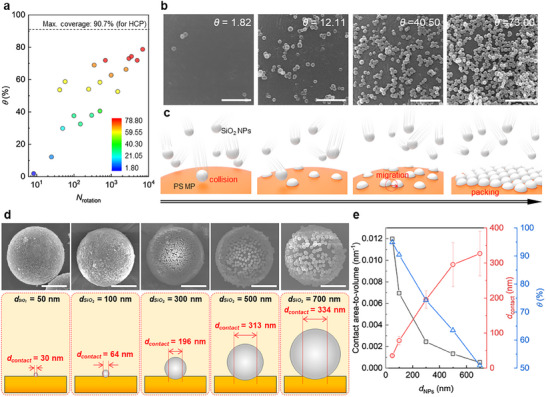
Mechanophysical assembly behaviors. a) The measured surface coverage (*θ*) of SiO_2_ NPs from the SiO_2_/PS HSPs as a function of the number of rotations (*N*_rotation_). b) SEM images of the SiO_2_ NPs assembled onto the PS MP as a function of *θ*, which was calculated as 1.82, 12.11, 40.50, and 73.00, respectively. c) Schematic illustrations of the mechanophysical synthesis processes of the SiO_2_ NPs onto the PS MPs. d) SEM images and the corresponding schematics of the SiO_2_/PS HSP as a function of the diameter of SiO_2_ NPs (*d*_SiO2_), ranging from 50 to 700 nm. e) Correlation plots of the contact area‐to‐volume ratios, *d*_contact_, and *θ* of the SiO_2_ NPs on the PS microsphere as a function of the diameter of SiO_2_ NPs. The scale bars denote 1 µm for (b) and 5 µm for (d).

Mechanophysical synthesis can be controlled by adjusting the mixing velocity and the collision time. Using the defined *N*_rotation_, the surface coverage (*θ*) and the number of adhered particles (*N*_particle_) can be determined by analyzing the guest NPs covering the specific region (3.4 × 4.9 µm^2^) of the host PS MP surface, as shown in **Figure** **3**a (Figure S21 and Table S5, Supporting Information).^[^ ^40^, ^41^ ^]^ When the collisions are insufficient (*N*_rotation_ < 10), only a few SiO_2_ NPs adhere on the PS MP surface, exhibiting low *d*_contact_ (Figure 2a). As *N*_rotation_ increases, *θ* logarithmically rises to ≈78.7%, nearing the theoretical maximum coverage of 90.7% with hexagonal closed packing (HCP). A similar logarithmic increase is also observed in *N*_particle_ (Figure S22, Supporting Information).

To further analyze the assembly behavior of SiO_2_ NPs on the PS MP, we examined the adhered NPs at specific *θ* values (Figure 3b). At *θ* = 1.82%, only a small number of SiO_2_ NPs adhere on the PS MP surface, and at *θ* = 12.11, the number of adhered NPs increases, with a few NP clusters formed due to cohesion between NPs. As *N*_rotation_ increases, the NPs progressively occupy the empty surface of the PS MP. Finally, the assembly of NPs on the MP surface becomes self‐limiting, as the NPs preferentially adhere to the remaining open areas of the MP. Combining the results from simulation (Figure 2) and experiments (Figure 3), we propose the mechanism for HSPs formation under the assumption that sufficient adhesion of the guest NPs is achieved in a high‐velocity environment. As illustrated in Figure 3c: i) At a low *N*_rotation_, collisions occur randomly around the surface of the PS MP, resulting in a small contact area that maintains the interfacial energy balance. As a result, only a few particles are slightly embedded. ii) As *N*_rotation_ increases, the guest NPs continue to collide with other particles and are pushed aside rather than embedding deeply into the host MP in the plane‐normal direction. This behavior is attributed to the lower density and *E* of the PS near the surface compared to the bulk, caused by increased molecular mobility at the surface where fewer neighboring molecules restrict movement.^[^ ^42^ ^]^ It was also reported that NPs under high pressure rearrange to rebalance interparticle forces and form ordered structures.^[^ ^43^ ^]^ Consequently, the NPs can migrate and pack more efficiently on the PS MP surface, as evidenced by migration tracks observed in the SEM image (Figure S23, Supporting Information). Finally, through repetitive collision and migration, the guest NPs fill the MP surface, achieving a *θ* of ≈86% by through a self‐limiting process.

Considering the importance of the contact area between guest and host particles in immobilizing the guest particles, we further investigated the assembly of the SiO_2_ NPs with different diameters (50, 100, 300, 500, and 700 nm) with the PS MPs (*d*_PS_ = 10 µm) under fixed conditions (700 RPM for 30 min) (Figure S24, Supporting Information). When smaller NPs (*d*_NP_, 50 nm or 100 nm) are mixed with PS MPs, the MP surface is entirely covered by NPs (Figure 3d). However, as *d*_NP_ increases, fewer NPs adhere to the MP surface due to their larger volume relative to the contact area. Consequently, the *θ* of SiO_2_ NPs decreases linearly with increasing *d*_NP_ (Figure 3e). To quantify the relationship between *d*_NP_ and *θ*, we further analyzed *d*_contact_ for HSP samples with varying NP diameters. As shown in Figure 3e, *d*_contact_ increased with the size of SiO_2_ NPs but was not directly proportional to *d*_NP_, aligning with simulation results (Figure S25, Supporting Information). This suggests that the effective contact area is limited by *d*_NP_. To explore this further, we evaluated the contact area‐to‐volume ratio of each SiO_2_ NP, which reflects the external force that can be borne  per unit area. The contact area was calculated as the hemispheric surface area of the PS MP in contact with an embedded single SiO_2_ NP (Figure 3e). The decrease in contact area‐to‐volume ratio indicates that the area receptible for contact on the PS MP surface decreases as *d*_NP_ increases, consistent with the observed trend of decreasing *θ*. During the collision, the large volume of the guest particles requires higher energy to deform the PS surface. Therefore, the SiO_2_ NPs with larger volume (or *d*_NP_) exhibit poorer adhesion to the PS MP surface, resulting in lower *θ* values as *d*_NP_ increases.

## 5 Multivalent Assembly of HSPs

**Figure 4:**
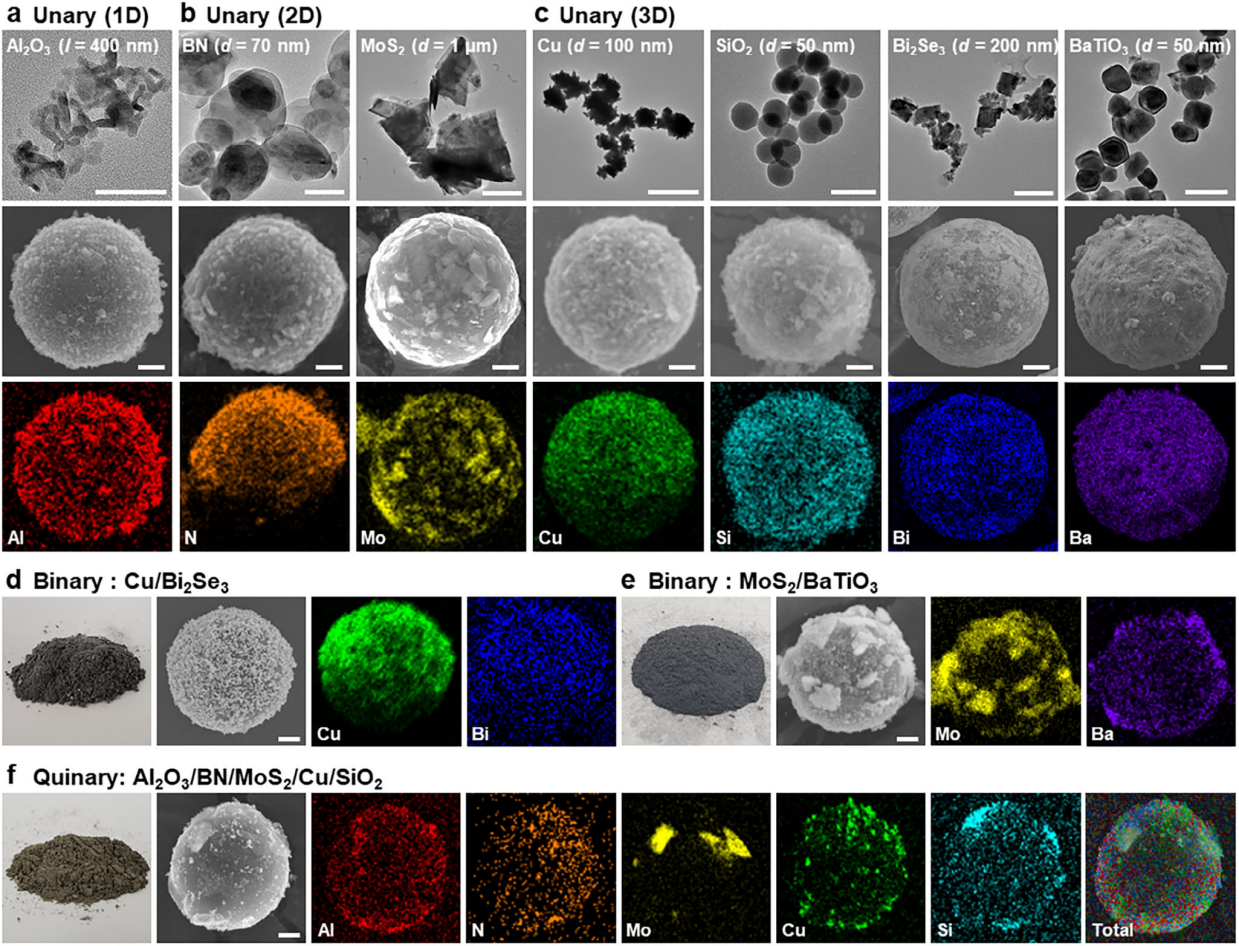
Single‐to‐multiple assemblies of heterologous particles. TEM images, SEM images, and EDS elemental mapping images of HSPs with a) 1D (Al_2_O_3_ fibers), b) 2D (BN, MoS_2_ platelets), and c) 3D (Cu, SiO_2_, Bi_2_Se_3_, and BaTiO_3_ particles) unary particles as a dimension of the particles, respectively. Morphological results as a combination were also shown in d) 3D/3D (Cu/Bi_2_Se_3_) and e) 2D/3D (MoS_2_/BaTiO_3_) binary mixture, and f) 1D/2D/3D (Al_2_O_3_, BN, MoS_2_, Cu, SiO_2_) quinary mixture, respectively. The scale bars denote 50 nm (Al_2_O_3_), 100 nm (BN, SiO_2_, Bi_2_Se_3_, and BaTiO_3_), and 500 nm (MoS_2_ and Cu) for (a‐c) (top row), 2 µm for (a‐c) (second row), and 2 µm for (d–f).

The synthesis of SiO_2_/PS HSPs is achieved by the large disparity in *d* (*d*_PS_: 10 µm, *d*_SiO2_: 100 nm) and *E* between the PS MPs and SiO_2_ NPs. In fact, the *E* of most inorganic materials is more than tens of times that of PS (Table S6, Supporting Information). To verify the universality of the mechanophysical synthesis method, we synthesized HSPs from different types of inorganic NPs with different dimensions and sizes (**Figure** **4**a–c): 1D aluminum oxide (Al_2_O_3_) nanofibers (NFs) with a diameter of 2–6 nm and length of 200–400 nm; 2D boron nitride (BN) and molybdenum disulfide (MoS_2_) nanosheets (NSs) with a diameter of 70 nm and 1 µm, respectively; and 3D copper (Cu), SiO_2_, bismuth selenide (Bi_2_Se_3_) and barium titanate (BaTiO_3_) NPs with a diameter of 100, 50, 200, and 50 nm, respectively. These NPs can impart new functionalities to the PS MP, such as heat resistance, thermal conductivity, dielectric properties, and semiconductor characteristics. All NPs are successfully loaded onto the host PS MPs regardless of their dimensionalities and sizes (Figure S26, Supporting Information). The exterior colors of as‐synthesized HSPs vary depending on the intrinsic properties of the guest materials. The uniform distribution of the guest NPs onto the host MPs is also confirmed by the appearance of the characteristic elements from the energy‐dispersive X‐ray spectroscopy (EDS) elemental mapping results.

Besides assembly of a single guest material, we synthesized HSPs with multiple guest materials simultaneously assembled onto the same PS MP. As shown in Figure 4d,e, when we employ the 3D Cu and Bi_2_Se_3_ NPs, a well‐mixed NP shell layer is observed on the MP surface. Notably, the Cu and SiO_2_ NPs, which have a large difference in density, were successfully loaded on the MP surface, and the resulting composition closely retained the initial input ratio, as confirmed by ICP‐OES (Figure S27 and Table S7, Supporting Information). In addition, the MoS_2_ NSs and BaTiO_3_ NPs with different dimensions can be loaded on the PS MP. We further demonstrated the quinary hybridization of Al_2_O_3_ NFs, BN NSs, MoS_2_ NSs, Cu NPs, and SiO_2_ NPs onto PS MPs (Figure 4f). Each of NFs, NSs, and NPs can successfully coexist on the MP surface, indicating that the mechanophysical synthesis strategy allows for the simultaneous imprinting of multiple functions of interest onto the PS MP surface regardless of composition, dimensions, and shapes of the nanomaterials.^[^ ^44^ ^]^

## 6 Synthesis and Application of Multi‐Functional HSPs

**Figure 5:**
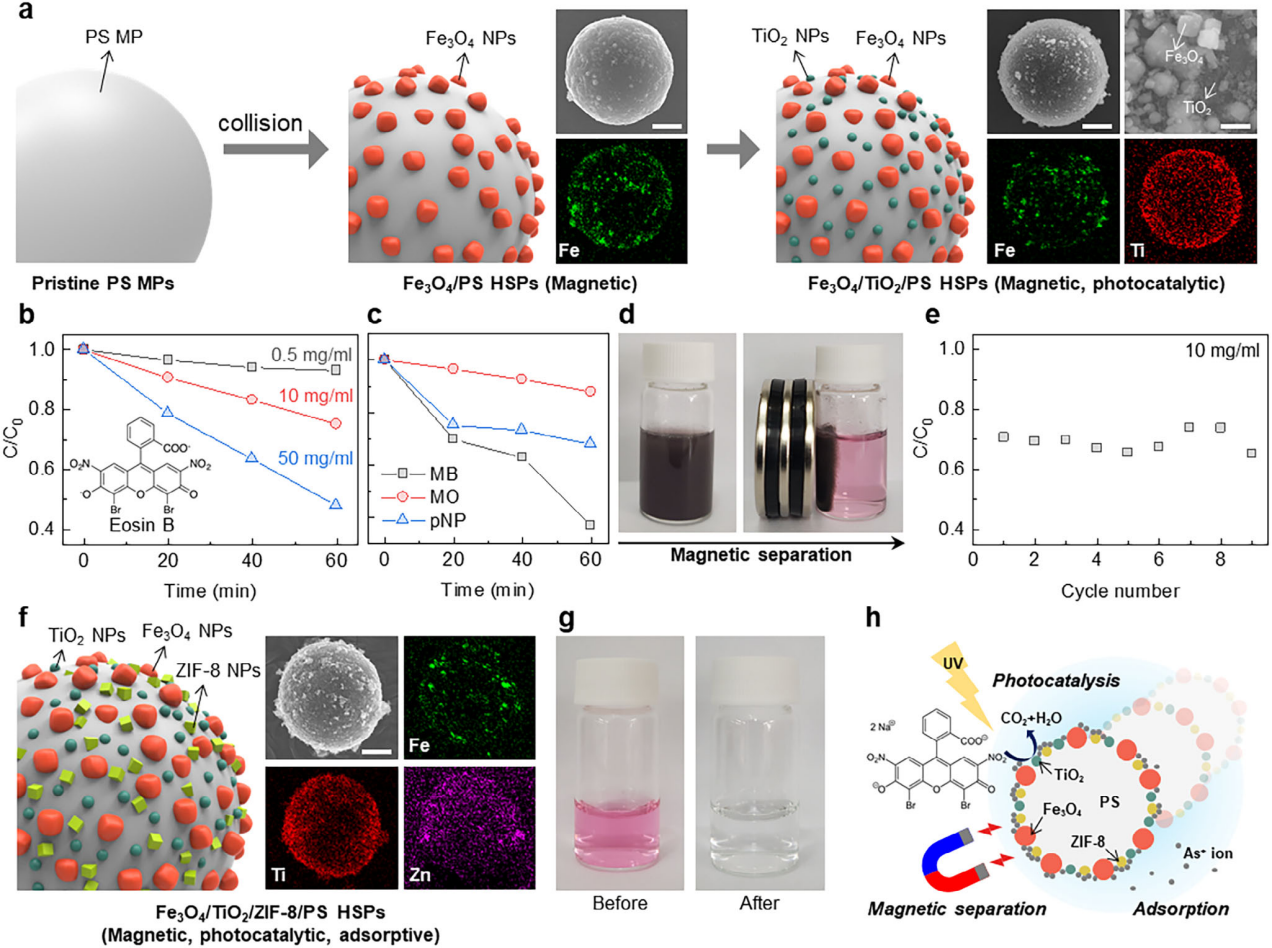
Magnetically separable HSP catalyst. a) Schematic illustration of the fabrication process for Fe_3_O_4_/PS HSPs and TiO_2_/Fe_3_O_4_/PS HSPs through mechanophysical assembly. In the EDS images, the green and red colors corresponded to the Fe and Ti elements. b) Relative concentration (*C*/*C_0_*) of the eosin B as a function of the HSP concentration and irradiation time. c) Relative concentration (*C*/*C_0_*) of the methylene blue, methyl orange, and *p*‐nitrophenol as a function of the irradiation time. d) Photographs of the magnetically separable HSPs. e) Resuability of the TiO_2_/Fe_3_O_4_/PS HSPs for nine cycles of the photocatalytic degradation test of the eosin B. f) Schematic illustration of fabricating Fe_3_O_4_/TiO_2_/ZIF‐8 HSPs. In the EDS images, the violet corresponds to the Zn element. g) Photograph of the As(III) solution before and after treatment by the HSPs. h) Schematic illustration of the multifunctional HSP showing simultaneously magnetic, photocatalytic, and adsorptive properties in a single particle. Scale bars denote 3 µm for (a‐f), and 400 nm for the magnified image in (a).

The ability to load a variety of hard NPs on the soft polymer MPs allows us to create HSPs that can simultaneously exhibit multiple functionalities.^[^ ^45^, ^46^ ^]^ First, we hybridized ferromagnetic Fe_3_O_4_ NPs to PS MPs (Figure 5a). It is noted that we employed vortex mixing for the collision to avoid stocking with a magnetic stirrer due to the magnetism of Fe_3_O_4_ NPs. After vigorous vortex mixing, the Fe_3_O_4_ NPs with a diameter of ≈500 nm are loaded on the surface of the PS MPs. Thus, the Fe_3_O_4_/PS HSPs with magnetic properties were successfully fabricated via collisions (Figures S28 and S29, Supporting Information).

To impart photocatalytic properties to the magnetic HSPs, TiO_2_ NPs, a well‐known photocatalyst, are hybridized with the magnetic Fe_3_O_4_/PS HSPs. The TiO_2_ NPs are loaded between the sparsely distributed Fe_3_O_4_ NPs on the PS MP surface, leading to the successful synthesis of Fe_3_O_4_/TiO_2_/PS HSPs that are simultaneously magnetic and photocatalytic (**Figure** **5**a; Figure S28, Supporting Information). Accordingly, we demonstrated photocatalytic degradation of eosin B, a well‐known dye pollutant using HSPs with a composition of 9.0 wt.% Fe_3_O_4_/0.9 wt.% TiO_2_/90.1 wt.% PS by considering finely covering the PS MP surface with NPs (Figure S30, Supporting Information). When the HSPs are put into the solution with eosin B of varying concentrations and exposed to UV light, eosin B molecules are disassociated into CO_2_ and H_2_O by TiO_2_ photocatalytically as a function of time (Figure 5b; Figure S31, Supporting Information). Likewise, methylene blue (MB), methyl orange (MO), and p‐nitrophenol (pNP) can be photocatalytically degraded by TiO_2_ (Figure 5c). Since Fe_3_O_4_/TiO_2_/PS HSPs also exhibit magnetism, they can be recovered using a magnet after degradation reactions with 100% yield (Figure 5d). None of these concepts is new; however, the preparation of multifunctional catalysts often involves complex processes, such as chemical assembly or in situ reduction on catalyst surfaces through wet chemistry. The mechanophysical synthesis demonstrated here significantly simplifies the fabrication. Finally, the magnetically separable Fe_3_O_4_/TiO_2_/PS HSPs can be recovered, washed, and reused, and exhibit stable photocatalytic degradation performance without detachment of the NPs from the MPs for at least 9 cycles (Figure 5e; Figure S32, Supporting Information).

We further hybridized HSPs with the ZIF‐8 NPs, a porous metal–organic framework, which is of interest for capturing a wide range of substances, from gas molecules to heavy metal ions, in addition to magnetic and photocatalytic properties. After vortex mixing all particles, the Fe_3_O_4_, TiO_2_, and ZIF‐8 NPs are successfully loaded on the surface of the PS MPs, where the Fe element in Fe_3_O_4_ NPs, Ti element in TiO_2_ NPs, and Zn element in ZIF‐8 NPs can be clearly seen in EDS (Figure 5f; Figure S28, Supporting Information). The HSP still possessed obvious magnetic properties due to the coexistence of the Fe_3_O_4_ NPs (Figure S29, Supporting Information). Using the Fe_3_O_4_/TiO_2_/ZIF‐8/PS HSPs, we target the removal of As(III) ions, a highly toxic heavy metal ion classified as a Group 1 carcinogen by the International Agency for Research on Cancer (IARC). Because the ZIF‐8 NPs are known for their exceptional As(III) ion removal capabilities, the multi‐functional HSPs effectively removed As(III) ions from solution through physisorption with van der Waals interactions within their unique nanoporous structure as well as chemisorption through interactions with Zn^2+^ ions at the active sites on the ZIF‐8 NPs surfaces (Figure 5g; Figure S33, Supporting Information). The Fe_3_O_4_/TiO_2_/ZIF‐8/PS HSPs can also be separated from the solution using a magnet.

Consequently, the multi‐functional HSPs with magnetic, photocatalytic, and adsorptive properties facilitate not only the effective degradation and removal of the pollutants based on different working principles using a single particle, but also easy recovery of the HSPs after utilization (Figure 5h).

## 7 Conclusion

We present a new concept to synthesize HSPs by colliding hard inorganic guest NPs with the soft polymer MPs through high energy impact during mechanical dry mixing at room temperature. During the collision, the surface of polymer MP is deformed, leading to a stable attachment of NPs on the craters of the MP without the need for wet chemistry. The assembly process can be precisely controlled by adjusting the surface energy of the NPs and the velocity of collision, thereby modulating the impact energy. A variety of multivalent HSPs are obtained by simultaneously incorporating two or more inorganic NPs, regardless of their types, shapes, or dimensions, onto the same MP. Accordingly, we successfully fabricated multi‐functional HSPs by sequentially introducing Fe_3_O_4_, TiO_2_, and ZIF‐8 NPs onto PS MPs, thereby endowing the co‐existence of magnetic, photocatalytic, and ion adsorptive properties on HSPs for removal of harmful pollutants and magnetic recovery after utilization. We envision the mechanophysical synthesis presented here offers a universal route for fabricating multiscale and multifunctional hybrid particles that can be customized on demand.

## Conflict of Interest

The authors declare no conflict of interest.

## Supporting information



Supporting Information

## Data Availability

The data that support the findings of this study are available from the corresponding author upon reasonable request.
