# Supplementary material for: Mechanophysical Synthesis of Core/Shell Hybrid Supraparticles
Source: Adv Mater. 2025 Apr 24;37(28):2502718. doi: 10.1002/adma.202502718 (PMC12271984; doi:10.1002/adma.202502718)
Supplement: Supplementary file 1 — Supporting Information [file ADMA-37-2502718-s001.pdf]

ADVANCED MATERIALS

Supporting Information

for *Adv. Mater.*, DOI 10.1002/adma.202502718

Mechanophysical Synthesis of Core/Shell Hybrid Supraparticles

Jeonguk Hwang, Seong Hwan Lee, Jinsu Kim, Geonho Lee, Jinwoo Park, Yunseok Choi, Jinhoon Lee, Jin Hong Lee, Jae Ryung Choi, Cheol-Min Yang, Il Jin Kim, Bo-In Park, Shu Yang, Seung-Yeol Jeon*, Dong Woog Lee* and Seunggun Yu**

Supporting Information

Mechanophysical Synthesis of Core/Shell Hybrid Supraparticles

Jeonguk Hwang, Seong Hwan Lee, Jinsu Kim, Geonho Lee, Jinwoo Park, Yunseok Choi, Jinhoon Lee, Jin Hong Lee, Jae Ryung Choi, Cheol-Min Yang, Il Jin Kim, Bo-In Park, Shu Yang, Seung-Yeol Jeon*, Dong Woog Lee*, Seunggun Yu**

Note 1. Methods

Materials: PS microparticles (MPs) were purchased from Microbeads AS (Norway). SiO_2 nanoparticles (NPs) were purchased from Sukgyung AT (South Korea). Aluminum oxide (Al_2O_3) nanofiber, barium titanate ($BaTiO_3$) NPs, iron oxide (Fe_3O_4) NPs, titanium oxide (TiO_2) NPs (anatase), methyl orange, zinc nitrate hexahydrate ($Zn(NO_3)_2 \cdot 6H_2O$), 2-methylimidazole, methylene blue, methyl orange, *p*-nitrophenol, 3-aminopropyltriethoxysilane (APTES), and trimethoxy(3,3,3,-trifluoropropyl)silane (FTMS) were purchased from Sigma Aldrich (USA). Boron nitride (BN) NPs was purchased from Graphene Supermarket (USA). Molybdenum disulfide (MoS_2) NPs were purchased from ALB Materials (USA). Copper (Cu) NPs were purchased from ACS Material (USA). Eosin B, bismuth (Bi), and selenium (Se) were purchased from Thermo Fisher Scientific (USA). Sodium arsenate dibasic heptahydrate ($Na_2HAsO_4 \cdot 7H_2O$), ethanol (EtOH), methanol (MeOH), isopropyl alcohol (IPA) was purchased from Daejung Chemicals and Metals (South Korea).

Bi_2Se_3 NPs were synthesized according to literature methods.[1] In a glove box, elemental bismuth and selenium powders were mixed in a stoichiometric ratio. 5 g mixture of these powders was then placed into a stainless steel jar with 50 g of zirconia balls. This jar was then positioned in a planetary ball mill (Pulverisette 6, Fritsch GmbH, Germany) and mixed at 550 rpm to synthesize Bi_2Se_3 NPs.

Zeolitic imidazolate framework-8 (ZIF-8) metal organic framework (MOF) was synthesized according to literature methods,[2] $Zn(NO_3)_2 \cdot 6H_2O$ (2.975 g, 0.010 mol) and 2-methylimidazole (MeIm, 3.284 g, 0.040 mol) were initially dispersed in 150 mL of MeOH, respectively. Two MeOH solution were mixed and stirred for 24 h. The white precipitates were collected via centrifugation and washed with MeOH several times. The as-prepared ZIF-8 NPs was subsequently dried in vacuum oven at 60 ºC.

Mechanophysical synthesis of SiO_2/PS HSPs: 400 mg of the PS MPs (diameter, 10 μm) and 200 mg of SiO_2 NP powders (diameter, 100 nm) were weighed and placed in a 10-mL glass vial. The mixture was mixed by magnetic stirring at 700 rpm for 30 min at the room temperature for synthesis of SiO_2/PS HSPs. After synthesis, unbound the SiO_2 NPs were removed by sonication (UCP-10, JEIOtech) at 300 W for 5 min, followed by filtration through a cellulose membrane filter (Grade 1, ADVANTEC group, pore size: 6 μm). The filtered powder was dried in fume hood at room temperature for 12 h to remove residual solvent. To enable scale-up, the synthesis of the SiO_2/PS HSPs was carried out in a custom-

designed batch-type apparatus at 700 rpm for 30 minutes. The post-treatment procedure was identical to that described above. Different sized SiO_2 NPs (diameter, 50, 300, 500, and 700 nm) were also examined by the same manner.

Surface modification of the SiO_2 NPs and their assemblies to form HSPs: SiO_2 NPs were surface-modified via silanization using APTES or FTMS. SiO_2 NPs and IPA were added to a glass reactor equipped with a condenser, and the mixture was vigorously stirred at 70 °C for 30 min. Subsequently, APTES or FTMS (6 wt% of the SiO_2 NPs) was introduced into the reactor, and the reaction was maintained at 70 °C for 4 h. The resulting mixture was filtered and dried under vacuum at 70 °C for 8 h to obtain the surface modified NPs. They were then mixed with PS MPs of varying revolutions per minute (RPM) for 10 min to analyze their affinity and adhesion on PS MPs.

Synthesis of multivalent HSPs: HSPs of different combination of NPs and MPs, including Al_2O_3/PS, BN/PS, MoS_2/PS, Cu/PS, SiO_2/PS, Bi_2Se_3/PS, $BaTiO_3$/PS, Cu/Bi_2Se_3/PS, MoS_2/$BaTiO_3$/PS, and Al_2O_3/BN/MoS_2/Cu/SiO_2/PS HSPs were synthesized by simultaneously mixing each powder at 700 rpm for 10 min.

Synthesis of Fe_3O_4/TiO_2/ZIF-8 HSPs: Fe_3O_4/TiO_2/ZIF-8 HSPs were synthesized via vortex mixing because Fe_3O_4 NPs are magnetic responsive. Briefly, 0.1g Fe_3O_4 NPs, 0.01g TiO_2 NPs, 1g PS MPs, and 5 zirconia balls (diameter, 1 mm) were placed in a vial and mixed for 30 min on the vortex mixer (VM-10, WiseMix, Portugal). Subsequently, the zirconia balls were separated and the collected HSPs powders were filtered and washed with EtOH several times to remove unreacted Fe_3O_4 and TiO_2 NPs. The obtained Fe_3O_4/TiO_2/PS HSPs were dried in a vacuum oven at room temperature. To synthesize Fe_3O_4/TiO_2/ZIF-8 HSPs, 0.01g ZIF-8 NPs were added to the via containing 1g Fe_3O_4/TiO_2/PS HSPs and mixed by the same manner.

General characterization: The photographs of the powders were taken by a cell phone (Galaxy Z Flip 4, South Korea). The morphology, microstructure and elemental mapping of various NP powders and HSPs was analyzed by scanning electron microscopy (SEM, S-4800, Hitachi, Japan) equipped with an energy-dispersive X-ray spectroscopy (EDS, EMAX, Horiba, Japan) system at the acceleration voltage of 15 kV. To further investigate the morphology at higher resolution, the particles were dispersed in water using an ultrasonic bath sonicator for 15 minutes, then collected on a carbon-coated template grid for sampling.

Bright-field (BF) images of the collected particles were obtained using transmission electron microscopy (TEM) (Titan G2 Cubed, FEI/One View, Gatan) at accelerating voltages below 80 kV. For SEM observation, the desired samples were placed on the holder and coated with osmium using a sputter coater (HPC-1SW, Vacuum Device, Japan). Typical morphology of the HSPs was also analyzed using SEM and a differential interference contrast (DIC) image (DMi8, Leica, Germany). In order to observe the crater structure and size of the SiO_2/PS HSPs samples, the SiO_2 NPs were slightly detached by ultrasonic processor (VCX 750, Sonics & Materials, USA) or fully etched immersing the samples in 1M NaOH aqueous solution. The crater size was calculated as an average from 10 measurements using the software ImageJ (https://imagej.net/ij/). The image processing procedure was used to enhance contrast and isolate the craters with image binarized from SEM images. The adhesion between the SiO_2 NPs and PS MP was analyzed by TEM (JEM-2100F, JEOL, Japan) at acceleration voltage of 200 kV. For TEM observation, the HSP powder was placed onto a Cu grid. The compositions in the HSPs were quantified using inductively coupled plasma optical emission spectroscopy (ICP-OES, 5900 ICP-OES system, Aigilent Technologies, US) equipped with a VistaChip II CCD detector. Elemental concentrations of Cu and Si were calculated using calibration curves and applied dilution factors. The magnetic properties of the Fe_3O_4 NPs and HSPs were characterized by vibrating sample magnetometry (VSM, LakeShore 7400) at the magnetic field of 10kOe at 300 K. The FT-IR spectra were collected by FT-IR (FT/IR-4200, Jasco, Japan) in the range of 4,000–400 cm^{-1} with 32 scans and a resolution of 4 cm^{-1}.

Temperature stability test: 200 mg of SiO_2/PS HSPs and 10 mL of distilled water were placed in a 10 mL glass vial. The vial was subjected to heating at 80 °C and the mixture was extracted at 30 min, 1 h, 6 h, 12 h, and 24 h, respectively. After heating, the mixture was vacuum filtered using a qualitative filter paper (1 grade, pore size: 6 μm, diameter, 55 mm, ADVANTEC group) and washed three times with EtOH. The filtrated samples were dried in a fume hood at room temperature for 12 h to remove any residual solvent.

Chemical stability test: 200 mg of SiO_2/PS HSPs and 10 mL of solvent or aqueous solution (EtOH, acetone, 10% HCl, 10% NaOH) were placed in a 10-mL glass vial. The mixture was subjected to magnetic stirring at 700 rpm for 24 h at room temperature for the evaluation of durability on solvent or pH. After stirring, the mixture was vacuum filtered using a qualitative filter paper (1 grade, pore size: 6 μm, diameter, 55 mm, ADVANTEC group) and washed

three times with EtOH. The filtrated samples were dried in a fume hood at room temperature for 12 h to remove any residual solvent.

Statistical analysis: MATLAB (v.R2024a) was used to calculate the statistical significance of comparison. Data distribution was assumed to be normal for all parametric tests but was not formally tested. The homogeneity of variance was confirmed through F-test between two samples groups. The two-sided Student's t-test was conducted to compare two sample groups with homoscedasticity.

Surface energy analysis: To analyze the adhesion behaviors, we prepared Si wafers (Silicon Technology Corporation, Japan) with different surface chemistries, including the pristine SiO_2, SiO_2-NH_2, and SiO_2-F. The Si wafers were cleaned, followed ultrasonic cleaning in ethanol and blow drying using a N_2 gas. The as-cleaned Si wafers was used as the pristine SiO_2 sample.

The SiO_2-NH_2 and SiO_2-F samples were prepared by treating the clean Si wafers with the corresponding silane agents. To activate the hydroxyl group on the surface of the Si wafers, the Si wafers was treated by O_2 plasma (CUTE, Femto Science, South Korea) at a radio frequency power of 100 W. For the SiO_2-NH_2 sample, the surface-activated Si wafers was immersed in a 2 v/v% APTES solution in ethanol for 60 min at the room temperature, followed by heat at 110 ºC for 15 min and then cooled to the room temperature. For the SiO_2-F sample, the surface-activated Si wafers was placed on top of a petri dish with a few drops of the FTMS solution in a desiccator, and then exposed to the FTMS vapor under vacuum for 2 h. After exposure, the Si wafers was cured at 110 ºC for 15 min and then cooled to the room temperature. Finally, the water contact angles of the pristine SiO_2, SiO_2-NH_2, and SiO_2-F samples were measured using a contact angle analyzer (Phoenix 300T, SEO), and the surface energy was calculated based on the Owens-Wendt method, which separates the total surface energy into polar and dispersive components.[3] This method involves measuring the contact angles of two probe liquids, typically polar (e.g., water) and nonpolar (e.g., diiodomethane) on the surface of interest.

$$\frac{\gamma_L(\cos\theta+1)}{2(\gamma_L{}^D)^{1/2}} = (\gamma_S{}^P)^{1/2}\frac{(\gamma_L{}^P)^{1/2}}{(\gamma_L{}^D)^{1/2}} + (\gamma_S{}^D)^{1/2} \qquad \text{(S1)}$$

The symbol of γ means the surface tension of the wetting liquid or the surface energy of the solid; a subscript indicates liquid, L, or solid, S, the superscript indicates dispersive, D, or polar, P; θ denotes the contact angle between the liquid and solid. The surface energy values

calculated with the equation, surface tension of liquids (water (γ = 72.8 mN m^{-1}) and diiodomethane (γ = 50.8 mN m^{-1})), and measured contact angles. Additionally, we measured five contact angles at each condition to ensure reliable statistics.
The interfacial energy between the PS and each silica (γ_{PS-SiO_2}) can be calculated by using the Good-Girifalco equation.[4]

$$\gamma_{PS-SiO_2} = \gamma_{PS} + \gamma_{SiO_2} - 2\sqrt{\gamma_{PS} \times \gamma_{SiO_2}} \quad \text{(S2)}$$

The surface tension of polystyrene was fixed (γ_{PS} = 34 mN m^{-1}) at three different conditions, while the values of silica (γ_{SiO_2}) are various as the surface treatment.[5]

Lap shear tests: The lap shear tests were performed using a universal testing machine (WL2100C, Withlab, South Korea). First, a Si wafer (0.5 in × 0.5 in) was dropped with 100 μL of PS solution (25 wt% in a benzene), and another Si wafer was placed on top of the PS-coated Si wafer, followed by drying in a vacuum chamber overnight. An acrylic substrate with adhesive was adhered on both sides of the sample to perform lap shear tests at a crosshead speed of 1.3 mm/min at the room temperature according to ASTM D1002 (Figure S9a,b). The lap shear strength values were obtained on average of five samples.

Adhesion force measurement: The Atomic Force Microscopy (AFM, Park FX40, Parksystems, South Korea) was used to measure the adhesion force between PS and SiO_2. PS solutions (25 wt% in benzene) were spin-coated onto the cleaned Muscovite mica substrate (Grade #1, S&J Trading, USA) at 1,000 rpm for 60 s. The silica colloidal probe (CP-NCH-SiO-E-5, *d*=15 μm, Probes Inc., South Korea) was used as the AFM tip. The force between silica colloidal tip and PS layer was evaluated according to load force with a penetration depth of 100, 150, 200, 250, 300 and 350 nm, respectively (Figure S9c,d). During the force measurement, the approach and separation of the colloidal probe were performed a 0.3 μm s^{-1} at the room temperature. For each sample under each condition, at least 5 measurements were conducted to confirm the reproducibility.

Photocatalytic activity studies: 1 g of HSPs was dispersed in a 20 mL aqueous solution of eosin B with a desired concentration under magnetic stirring for 3 h.[6] The mixture was placed under a UV light with a wavelength of 260–280 nm (15W, Sankyo Denki, Japan) in a self-made darkroom, and mixed by an overhead mechanical stirrer (High-Torque Overhead Stirrer, Daihan Scientific, South Korea) at 150 rpm. After a certain period of time, 5 mL aliquot was taken and the magnetically responsive HSPs were isolated using Neodymium

magnet to stop the catalytic reaction. The amount of remaining eosin B after the photocatalytic degradation was determined by measuring the absorption intensity at the 521 nm using the UV-vis spectrometer (V-770, Jasco, Japan) based on a calibration curve made with a reference solution of eosin B. Photocatalytic degradation experiments of the methylene blue, methyl orange, and *p*-nitrophenol were performed by the same manner. For cycling tests, HSPs separated by the magnet were redispersed in the freshly-prepared eosin B solution and the photocatalytic degradation experiments were performed up to 9 cycles.

As(III) adsorption studies:1 g HSPs was dispersed into a 20 mL aqueous solution of sodium arsenate dibasic heptahydrate at a concentration of 0.1 mg mL^{-1}.[7] The mixture was mixed by an overhead mechanical stirrer (High-Torque Overhead Stirrer, Daihan Scientific, South Korea) at 150 rpm for 3 h. 5 mL aliquot was taken at 20, 40, and 60 min, respectively, and the magnetically responsive HSPs were separated using Neodymium magnet to inhibit further adsorption. The As(III) concentrations were measured by Inductively Coupled Plasma Optical Emission Spectrometry (ICP-OES, Optima 8300, Perkin Elmer, USA).

Note 2. Simulation Details

Correlation between ω and the impact velocity: To simulate particle collisions due to stirring as a one-dimensional collision, the following assumptions were made (Figure S9): 1) The kinetic energy of the rotating magnetic stirrer is fully transferred to the particles. Due to variations in the moment of inertia with rotation radius, particles receive different kinetic energies based on their position—those near the center receive less, while those farther out receive more. 2) At a rotation radius, the kinetic energy is transferred to the particles inversely proportional to their mass ratio, based on the law of conservation of momentum. 3) Most particles are within the magnetic stirrer's rotation radius, and those receiving kinetic energy move in a circular path around its center. 4) Energy losses from air drag, inter-particle friction, and higher-order collisions are neglected.

The simulated SiO_2/PS hybrid particle synthesis was performed using a cross-type magnetic bar (length: 10 mm, height: 5 mm), and the moment of inertia (*I*) of the magnetic bar is as follows:

$$I = \frac{2}{3}MR^2 \qquad \text{(S3)}$$

, where *M* is the mass of the magnetic bar (0.7 g), and *R* is the radius of the magnetic bar (*l*/2 = 5 mm).

The kinetic energy (*KE*) is, then, given by

$$KE = \frac{1}{2}I\omega^2 \qquad \text{(S4)}$$

, where ω is the angular velocity (rad s^{-1}), and its unit is equivalent to 60/2π rpm.

The total number of each particle (PS and SiO_2) within the rotation radius is calculated using the mass and density of each particle:

$$\#of\ PS\ particles = \frac{0.4\ g}{4/3\pi(5\ \mu m)^3 1050\ kg/m^3} = 7.27 \times 10^8 \qquad \text{(S5)}$$

$$\#of\ SiO_2\ particles = \frac{0.2\ g}{4/3\pi(50\ nm)^3 2650\ kg/m^3} = 1.44 \times 10^{14} \qquad \text{(S6)}$$

Assuming each particle is distributed within the rotation radius of the magnetic stirrer and follows a specific circular orbit around its center of rotation, the number of possible orbits is approximately *R* divided by the particle's diameter. For PS particles, this results in R/d_{PS} = 500, while for SiO_2 particles, it is R/d_{SiO2} = 50,000,000. Based on the rotation center of the magnetic stirrer, the particles are assumed to be distributed according to the following multinomial distribution, weighted by the rotation radius (*r*):

$$P(x_1, x_2, \cdots, x_k) = \frac{k!}{x_1! x_2! \cdots x_k!} p_1^{x_1} p_2^{x_2} \cdots p_k^{x_k} \cdot \left(\prod_{i=1}^{k} r_i^{\alpha x_i}\right) \qquad \text{(S7)}$$

where k is the number of possible orbits, p_i is the probability of a particle occupying each orbit, x_i is the number of particles in each orbit, r_i denotes the radius of each orbit, and α is the exponent that determines the weighting strength. Figure S10 presents the particle distribution based on the rotation radius, following the radius-weighted multinomial distribution.

Assuming that the kinetic energy of a magnetic stirrer at a rotation radius r is evenly transferred to the particles in orbits corresponding to r, the kinetic energy of each particle (KE_{PS} and KE_{SiO_2}) is:

$$KE_{PS} = \frac{\frac{1}{2}\left(\frac{2}{3}Mr^2\right)\omega^2}{\#\ of\ PS\ particles\ at\ r} = \frac{1}{2}(M_{PS}r^2){\omega_{PS}}^2 \quad \text{(S8)}$$

$$KE_{SiO_2} = \frac{\frac{1}{2}\left(\frac{2}{3}Mr^2\right)\omega^2}{\#\ of\ SiO_2\ particles\ at\ r} = \frac{1}{2}\left(M_{SiO_2}r^2\right){\omega_{SiO_2}}^2 \quad \text{(S9)}$$

, where M_{PS} and M_{SiO_2} represent the masses of a single PS and SiO_2 particle, respectively, and ω_{PS} and ω_{SiO_2} denote their angular velocities.

From the above equations, ω_{PS} and ω_{SiO_2} are calculated. By converting the angular velocity into linear velocity using the relation $\omega * r = v$, the relative velocity (v_{rel}) between a PS particle and a SiO_2 particle is as follows:

$$v_{rel} = abs(v_{PS} - v_{SiO_2}) \quad \text{(S10)}$$

, where v_{PS} and v_{SiO_2} represent the velocities of a single PS and SiO_2 particle.

According to the calculation results, when the magnetic stirrer rotates from 0 to 1500 rpm, the velocity of a PS particle reaches a maximum of 12.25 m s^{-1}, while the velocity of a SiO_2 particle increases to 7747.08 m s^{-1}. Due to the significant difference in velocities, it is suggested that the experimental situation can be simulated as a collision between SiO_2 particles and fixed PS particles. Figure S11 exhibits the relationship between the relative speed and the angular velocity (unit: rpm) of the magnetic stirrer as a function of the rotation radius (r), and demonstrates how it changes with the weight α. Although the relative velocity range varies depending on α, the average calculated relative velocity range is from 136.79 to 4103.78 m s^{-1}, which is approximately equivalent to 1 rpm ~ 2.64 m s^{-1}. The actual experimental results closely match the simulations at 1 rpm ~ 1 m s^{-1}, which is believed to be due to energy loss from inter-particle friction and higher-order collisions.

Governing equations: A numerical model depicting particle adhesion under various impact velocities is developed using COMSOL Multiphysics 6.2 (Burlington, MA, USA). The physical attraction between two particles can be described by:[8]

$$\rho \frac{d^2\boldsymbol{u}}{dt^2} = \nabla \cdot (FS)^T + F_B \tag{S11}$$

where ρ is mass density, $\boldsymbol{u}$ is the displacement tensor, t is time, F is the deformation gradient, S is the second Piola-Kirchhoff stress tensor, and F_B is a body force.

The guest NP (SiO_2) is assumed to follow a linear elastic model with Young's modulus and Poisson's ratio obtained from the COMSOL material database. The host microparticle (PS), on the other hand, is modeled as a neo-Hookean hyperelastic material, with parameter values determined from tensile tests. The material parameters utilized in the model are summarized in Table S2. Assuming that the hyperelastic material is nearly incompressible, the strain energy is given by:[9]

$$W = \frac{1}{2}\mu(I_1 - 3) + \frac{1}{2}\kappa(J - 1)^2 \tag{S12}$$

where μ is Lamé parameters, κ is bulk modulus, and I_1 is the first invariant given by:

$$I_1 = {\lambda_1}^2 + {\lambda_2}^2 + {\lambda_3}^2 \tag{S13}$$

where λ_i are the principal stretches and J is the Jacobian of the deformation gradient, $J = \det(F)$. The stress tensor S is expressed as the derivative of the inelastic stress (S_{inel}) and strain energy density (W) with respect to the strain:

$$S = S_{inel} + \frac{\partial W}{\partial \varepsilon} \tag{S14}$$

Since the diameter of the host particle is much larger than that of the guest particle (up to 100 times larger), the geometry of the host particle is modeled as a flat surface. The bottom boundary of the host particle is fixed. The initial velocity of the guest particle is prescribed in the form of a body force (Figure S12).

As the guest particle collides with and adheres to the host particle, it dissipates kinetic energy due to frictional and inelastic losses. To simulate this behavior, damping properties are incorporated into the host particle.[10–12] In this model, the particles adopt viscous damping properties, following the equation:

$$S_{inel} = \left(\eta_b - \frac{2}{3}\eta_v\right)\dot{J}C^{-1} + 2\eta_v J C^{-1}\dot{\varepsilon}C^{-1} \tag{S15}$$

where η_b is bulk viscosity and η_v is shear viscosity summarized in Table S2.

Contact modeling: Contact between host and guest particles is modeled using penalty formulation implemented in COMSOL Multiphysics 6.2. If the gap distance between the

boundaries of the guest and host particles, g, becomes negative (indicating that the guest particle boundary is penetrating the host particle boundary), the contact pressure in the normal direction is computed as[13]

$$T_n = \begin{cases} -f_p \frac{E_{char}}{h_{min}} g & if\ g \leq 0 \\ 0 & if\ g > 0 \end{cases} \quad \text{(S16)}$$

where f_p is the penalty factor multiplier, E_{char} is the characteristic stiffness assumed to be equal to the stiffness of the host particle, and h_{min} is the minimum element size on the contact pair.

The surface affinity between host and guest particles is taken into account adjusting f_p. Surface repulsion (weak surface attraction) is modeled with low f_p to account for the strong repulsion between two particles, which cancels out the contact pressure, thus lowering it upon contact. Conversely, when surface affinity is substantial, the resulting contact pressure between two particles is higher than when the attraction is weak, so this scenario is represented with large f_p.

Adhesion: Adhesion between two particles is activated when the contact pressure, T_n, exceeds a critical pressure, p_0. Upon activation, the adhesive layer formed when the two particles come into contact is considered a thin elastic layer, and the deformation of this layer with stiffness, k, results in adhesive stress. k is defined as[8]

$$\mathrm{k} = \{n_\tau p_n, n_\tau p_n, p_n\} \quad \text{(S17)}$$

where n_τ is the shear-to-normal ratio, assumed to be 0.2, and p_n represents $f_p E_{char}/h_{min}$. The adhesive stress is developed as $\mathrm{k}u_l$, where u_l is the displacement of the thin elastic layer. Two different critical pressures are applied to simulate a situation in which the interfacial energy between host and guest particles is dominant, and another situation where adhesion primarily occurs due to contact pressure resulting from dominant collision energy. In the latter case, p_0 is set to an infinitesimally small value, such as 10^{-8} Pa, to simulate adhesion dependent on collision energy. The case where interfacial energy is dominant is implemented by setting p_0= 0. In this scenario, adhesion occurs between the surfaces of the guest and host particles regardless of the magnitude of the contact pressure, resulting in the formation of adhesive stress even before collision.

Decohesion: Decohesion is considered using a cohesive zone model, in which all or part of the previously formed adhesion between two particles is removed when the collision energy is

significant, generating a large repulsive force between them. If the thin elastic adhesive layer deforms, exceeding critical traction (τ_c) and critical separation (δ_c), decohesion occurs, and traction is reduced until a critical separation (δ_f), at which the traction becomes zero (Figure S13). The traction (τ) generated by the deformation of the adhesive layer is as follows:

$$\tau = (1 - d)\frac{\tau_c}{\delta_c}\delta \quad \text{(S18)}$$

where d is the displacement-based damage variable defined such that $d = 0$ when the adhesive layer is undamaged and $d = 1$ when it is completely damaged, and δ is the separation.

To predict the damage of the adhesive layer under tensile (mode I) and shear (mode II) stresses, a power law criterion was employed as follows:

$$\left(\frac{G_I}{G_{Ic}}\right) + \left(\frac{G_{II}}{G_{IIc}}\right) = 1 \quad \text{(S19)}$$

where G_I, G_{II}, G_{Ic}, and G_{IIc} denote fracture energy release rates and critical fracture energy release rates, respectively.

To obtain the parameters required for the cohesive zone model, specimens were fabricated by bonding two Si wafers with PS. Shear strength and critical shear (fracture) energy release rate were measured from lap shear tests on these specimens (Figure S14a,b). Tensile properties were found to be values that were simulated to match the adhesion energy values obtained by loading and unloading SiO_2 particles onto the PS substrate using AFM (Figure S14c,d). The parameters used in the cohesive zone model are listed in Table S3.

As a result of simulating the loading and unloading of a guest particle on the surface of a host particle using the above parameters, it is confirmed that JKR (Johnson-Kendall-Robert)-like contact behavior (short-range adhesion) is observed, in which the surface of the host particle was somewhat dragged during unloading (Figure S15a). Meanwhile, when the tensile and shear strength are set to less than 1/20 of those that exhibit JKR-like contact behavior, DMT (Derjaguin-Muller-Toporov)-like contact behavior (long-range adhesion) is observed, with almost no surface drag (while the tensile and shear energy release rates are the same) (Figure S15b).

To consider realistic interactions between the guest particles and the host particles, the JKR-like contact condition is applied to the area where direct contact between the two particles occurs, and the DMT-like contact condition is applied to the outside contact. The region of the host particle, equal to or smaller than the radius of the guest particle, where direct contact between the guest particle and the host particle can occur, is designated as the JKR-like contact region. The remaining part is designated as the DMT-like contact region (Figure S16).

Parametric studies on the adhesion behaviors: The variation in contact diameter with particle impact velocity is investigated, considering three parameters: penalty factor (surface affinity, f_p), criterion pressure for adhesion activation (p_0) and the ratio of the JKR-like region to the radius of the guest particle (λ) (Figure S19). When considering the adhesive stress between two particles before collision by setting $p_0 = 0$, it is confirmed that when the JKR-like area is small, the guest particle is more deeply embedded in the host particle as the collision speed increases, resulting in an increased contact diameter. Meanwhile, it is found that as the JKR-like region becomes wider, the contact diameter tends to become constant regardless of the impact velocity. Additionally, as the surface affinity increases, this tendency becomes more pronounced, and the collision speed-independent contact diameter increases. The impact velocity independence occurs because the repulsion energy generated by the collision is not large enough to cause separation of the adhesive layer already formed between particles via collision. Furthermore, the greater the surface affinity, the more difficult for decohesion occur. On the other hand, In the case of $p_0 > 0$ where adhesion occurs solely due to collision, regardless of λ, the contact diameter increases with increasing collision speed and eventually converged. There is no significant difference in contact diameter depending on surface affinity, except in the case of low surface affinity (f_p = 0.1), where convergence of contact diameter occurs at a lower impact velocity, and no adhesion occurred at higher velocities after convergence.

Energy contribution to particle adhesion: When a guest particle collides with a host particle, the guest particle is maximally embedded immediately after the collision (Figure S18a). However, as time passes, the host particle's repulsion and the adhesion between the two particles result in an equilibrium with the guest particle being less embedded. At this time, the energy involved includes kinetic energy, elastic strain energy, contact energy, adhesive elastic energy, and dissipation, all of which change over time as shown in Figure S18b. Elastic strain energy arises from the elastic deformation of two particles, contact energy is the energy needed to establish a contact surface between them, and adhesive elastic energy is the energy resulting from the deformation of a newly formed thin elastic adhesive layer between the contact surfaces. Dissipation occurs due to the viscoelastic characteristics of the host particle. The kinetic energy of the guest particle decreases rapidly after collision and is converted into dissipation, elastic strain, contact, and adhesive elastic energies. In other words, the adhesion

of guest particles to host particles is maintained by balancing the remaining energies lost through dissipation of kinetic energy.

Modeling the transition of the adhesion mechanism: The simulation results confirm that the dependence of the contact diameter on impact velocity varies based on the criterion pressure (p_0) for adhesion activation. When examining the realistic contact behavior observed in experiments, it is found that at low collision speeds, a constant contact diameter is maintained regardless of the speed (similar to the simulation case with $p_0 = 0$). However, above a certain speed, the contact diameter increases with increasing speed (similar to the simulation case with $p_0 > 0$). To embody the contact behavior observed in experiments, specific criteria are needed for how to combine two different simulation cases and at what collision speed a transition can occur between the cases of $p_0 = 0$ and the case of $p_0 > 0$. Based on the simulated contact energy according to the impact velocity, if the case with the higher simulated contact energy is adopted between the two simulation cases, the impact velocity at which the transition between the two cases occurs can be set, as shown in Figure S17. It is confirmed that the simulation results match well with the actual experimental results when $p_0 = 0$ is used below this velocity and $p_0 > 0$ is used thereafter. Simulations are implemented for different particle cases using specific parameters: SiO_2-NH_2 particles with $f_p = 3$ and $\lambda = 1$; pristine SiO_2 particles with $f_p = 2.5$ and $\lambda = 0.6$; and SiO_2-F particles with $f_p = 1$ and $\lambda = 0.55$.

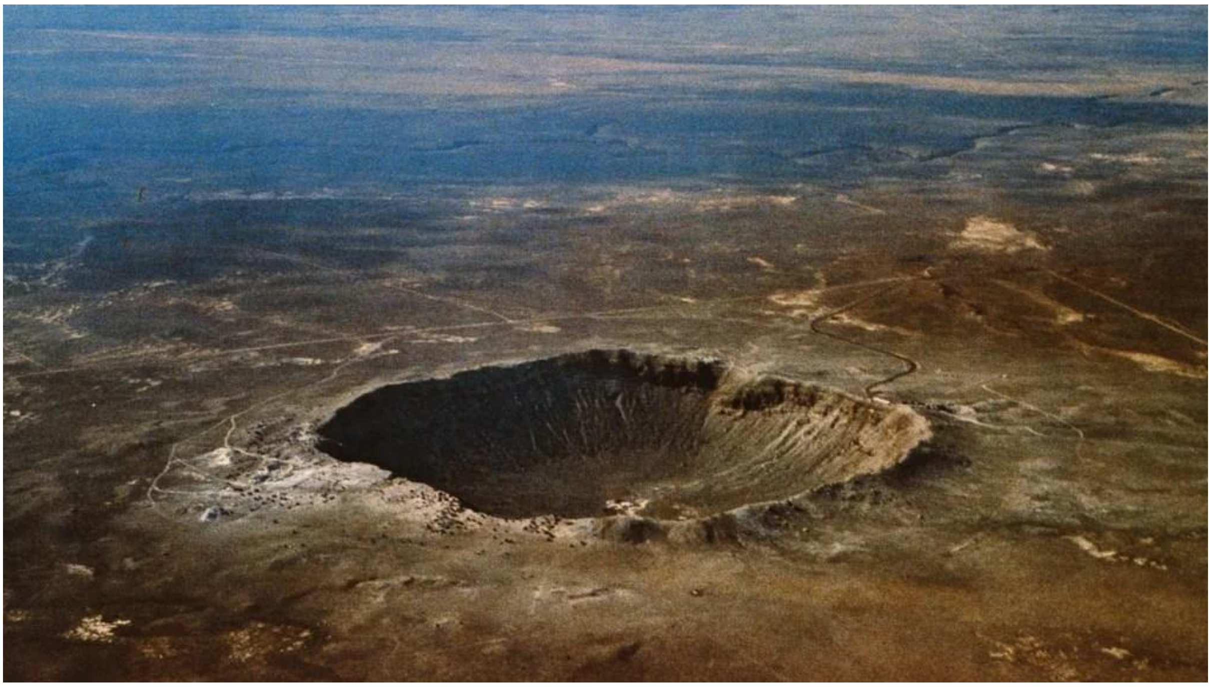

Figure S1. Photograph of the *Barringer Crater*. The photograph was taken by D. Roddy. (https://www.lpi.usra.edu/publications/slidesets/craters/slide_10.html)

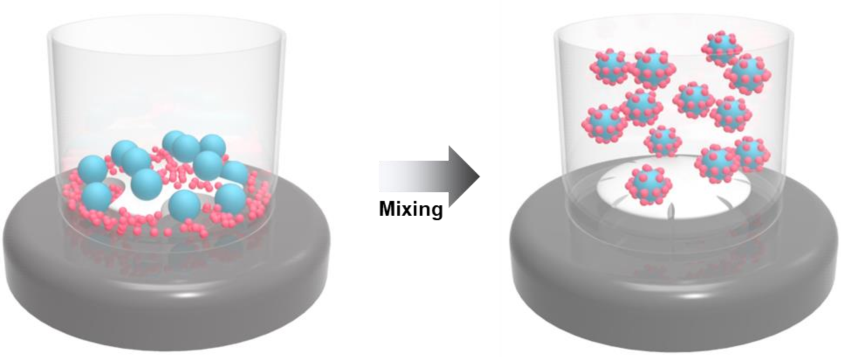

Figure S2. Schematic illustration of the HSP fabrication process through simple dry mixing.

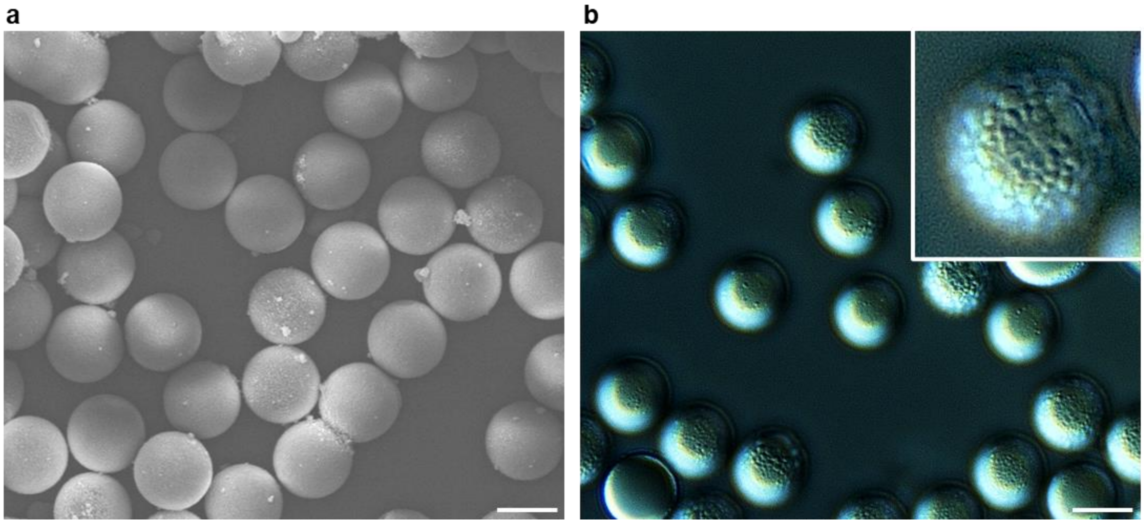

Figure S3. a,b) Low-magnification SEM and DIC images of the SiO_2/PS HSPs. The scale bars denote 10 μm.

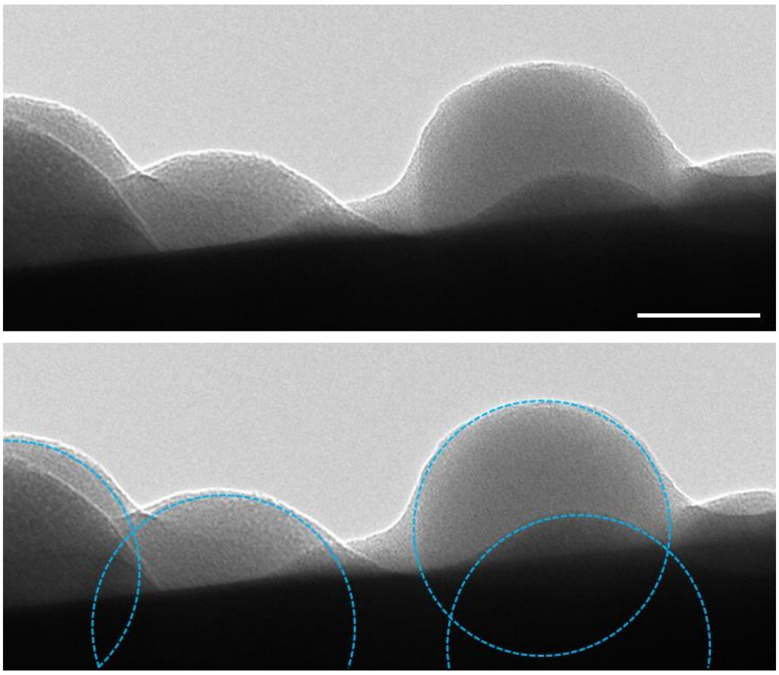

Figure S4. Morphology analysis of SiO_2/PS HSPs. HR-TEM image of SiO_2/PS HSPs, in which the interface between SiO_2 NPs and PS ridge was identified with contrast difference that was also highlighted using blue dotted line. The scale bar denotes 50 nm.

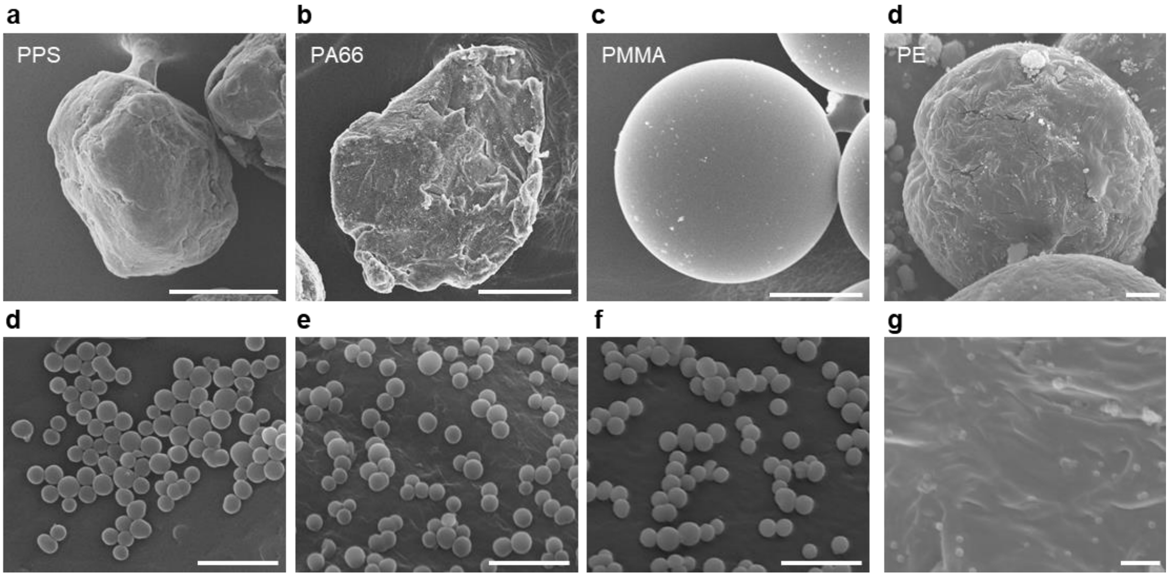

Figure S5. Demonstration of the attachment of SiO_2 NPs onto the surfaces of various polymer microparticles. SEM images of (a) PPS, (b) PA66, (c) PMMA, and (d) PE at low magnification and their corresponding high magnification (d–g). The scale bars denote 50 μm for (a–c), 5 μm for (d), 100 nm for (d–f), and 500 nm for (g).

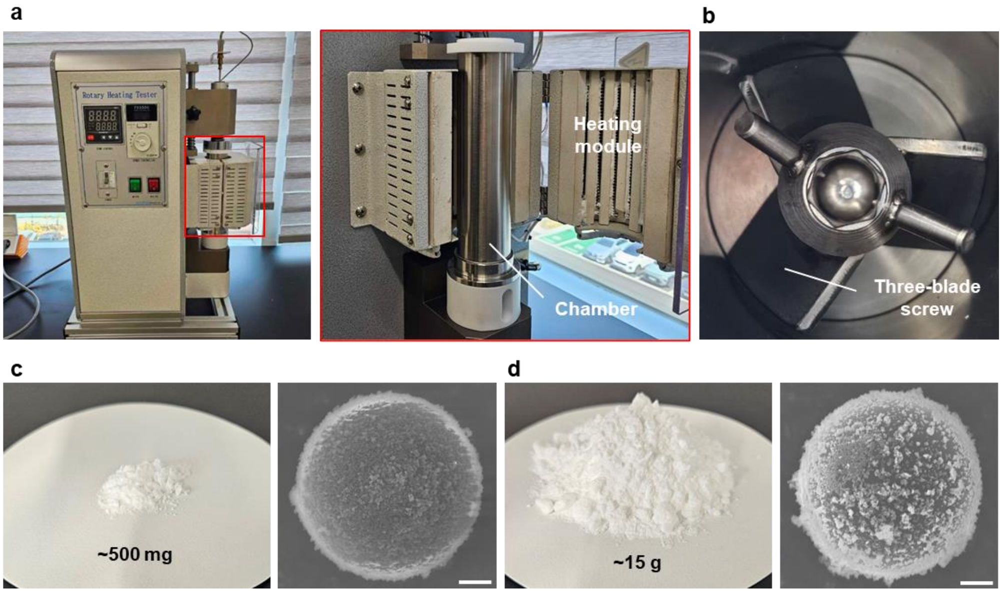

Figure S6. Custom-built apparatus for scale-up synthesis of HSPs. a) Photograph of the full apparatus, which is equipped with a motor-driven rotary system and a heating module for potential temperature-controlled operation. b) Internal view of the chamber showing the bottom-mounted three-blade screw-type impeller used for mixing the powder. Photograph and SEM images of the HSPs synthesized via magnetic stirring in a vial (c) and the scale-apparatus (d). The scale bars denote 2 μm.

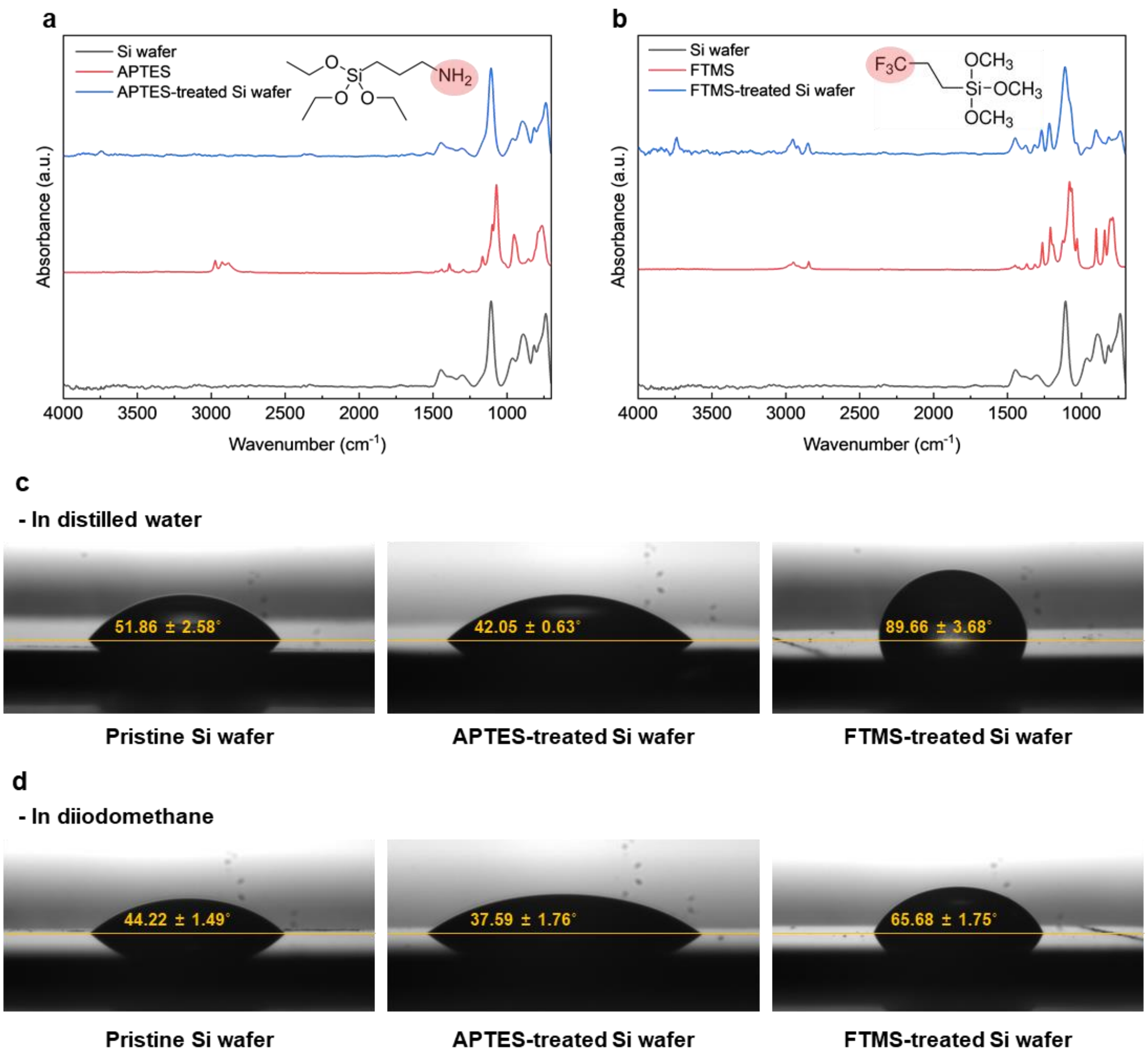

Figure S7. Surface analysis of the silane-functionalized a Si wafer with a native oxide layer. FT-IR spectra of (a) pristine Si wafer, APTES, and APTES-treated Si wafer, and (b) pristine Si wafer, FTMS, and FTMS-treated Si wafer. Optical microscopic images observing contact angle of (c) distilled water and (d) diiodomethane on the pristine Si wafer, APTES-treated Si wafer, and FTMS-treated Si wafer.

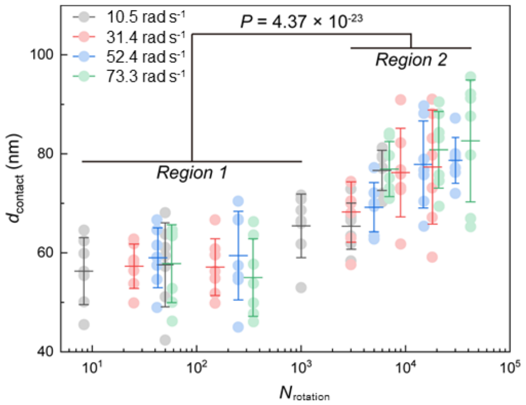

Figure S8. Contact diameter ($d_{contact}$) of SiO_2 NPs on PS MP as a function of the number of rotations ($N_{rotation}$). The middle line, whiskers, and circle dots represent the mean, standard deviation, and individual data points (n = 7), respectively.

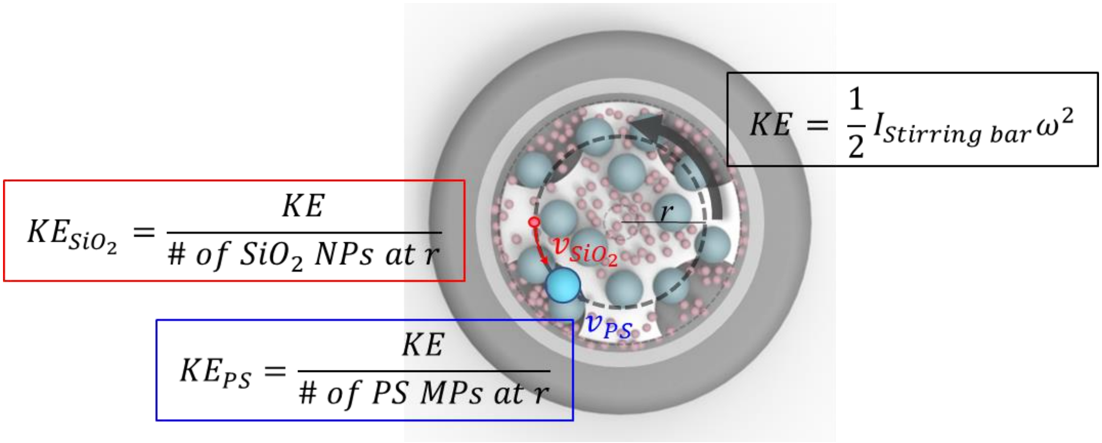

Figure S9. Schematic representation of assumptions for simulating particle collisions induced by magnetic bar stirring.

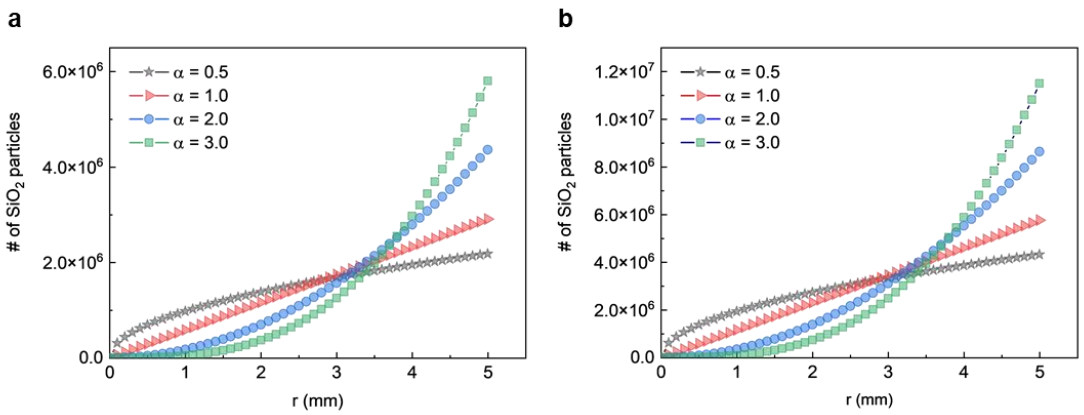

Figure S10. Distribution of particles at each orbit with increasing radius; (a) PS particles, and (b) SiO_2 particles.

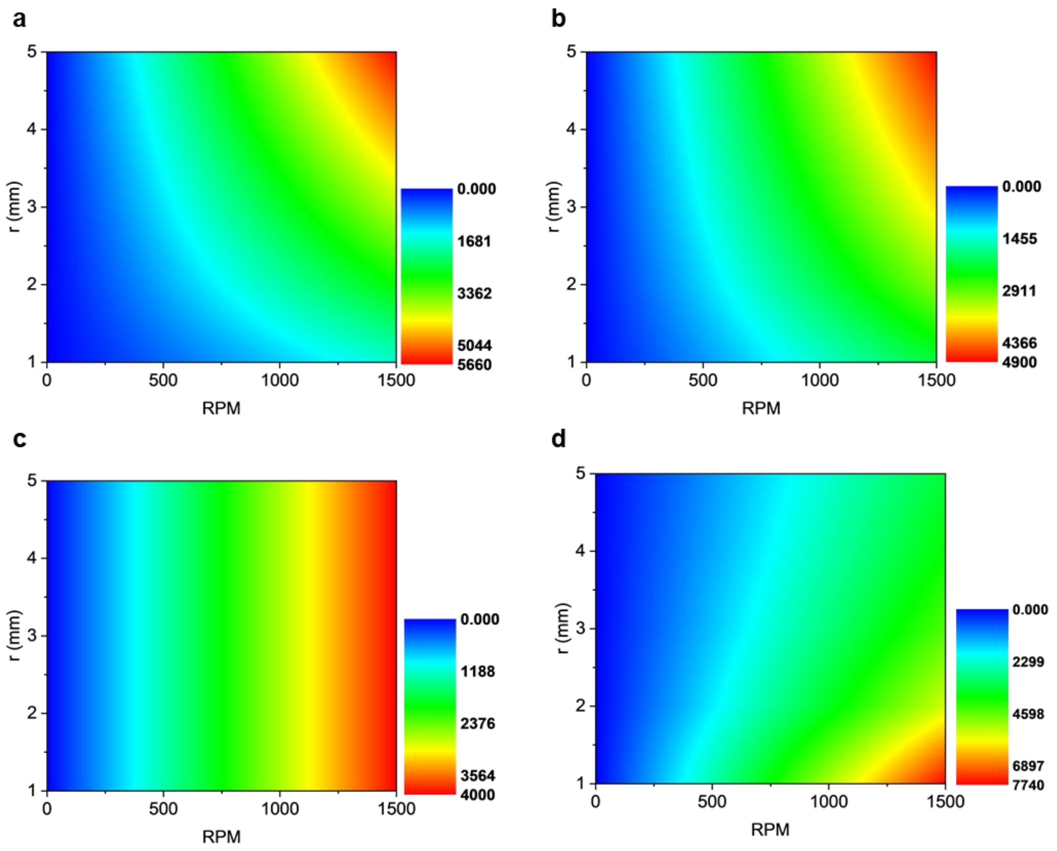

Figure S11. Relationship between relative speed (impact velocity) and angular velocity (RPM) of the magnetic stirrer as a function of rotation radius for (a) α = 0.5, (b) α = 1.0, (c) α = 2.0, and (d) α = 3.0.

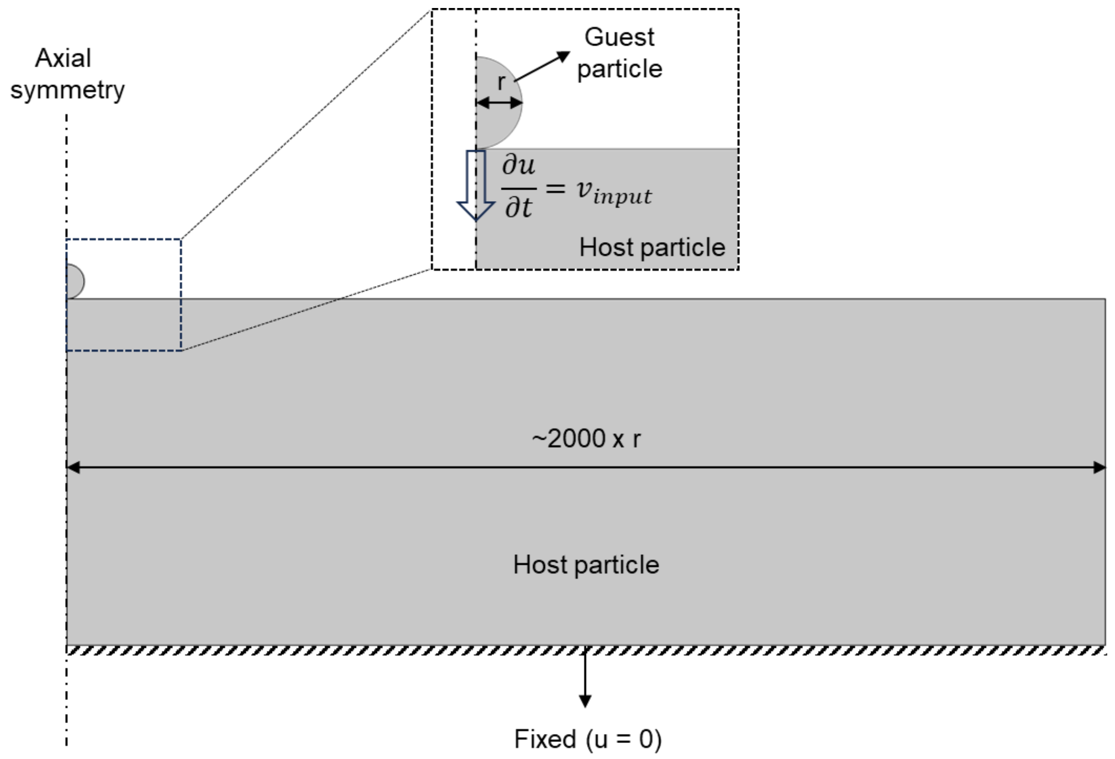

Figure S12. Geometry and boundary conditions assigned to the numerical model.

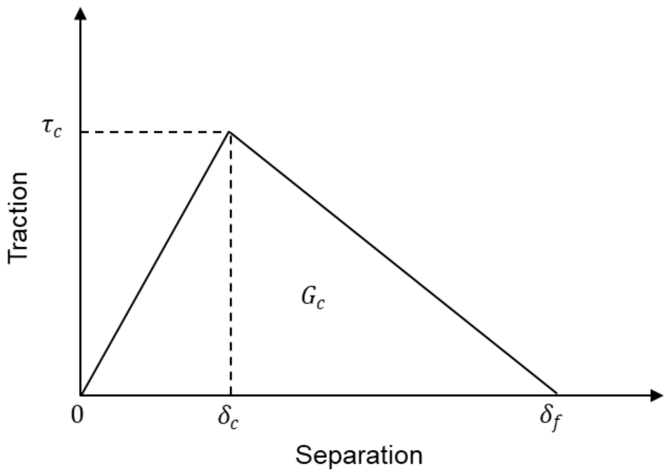

Figure S13. Bilinear traction-separation law in cohesive zone model for decohesion.

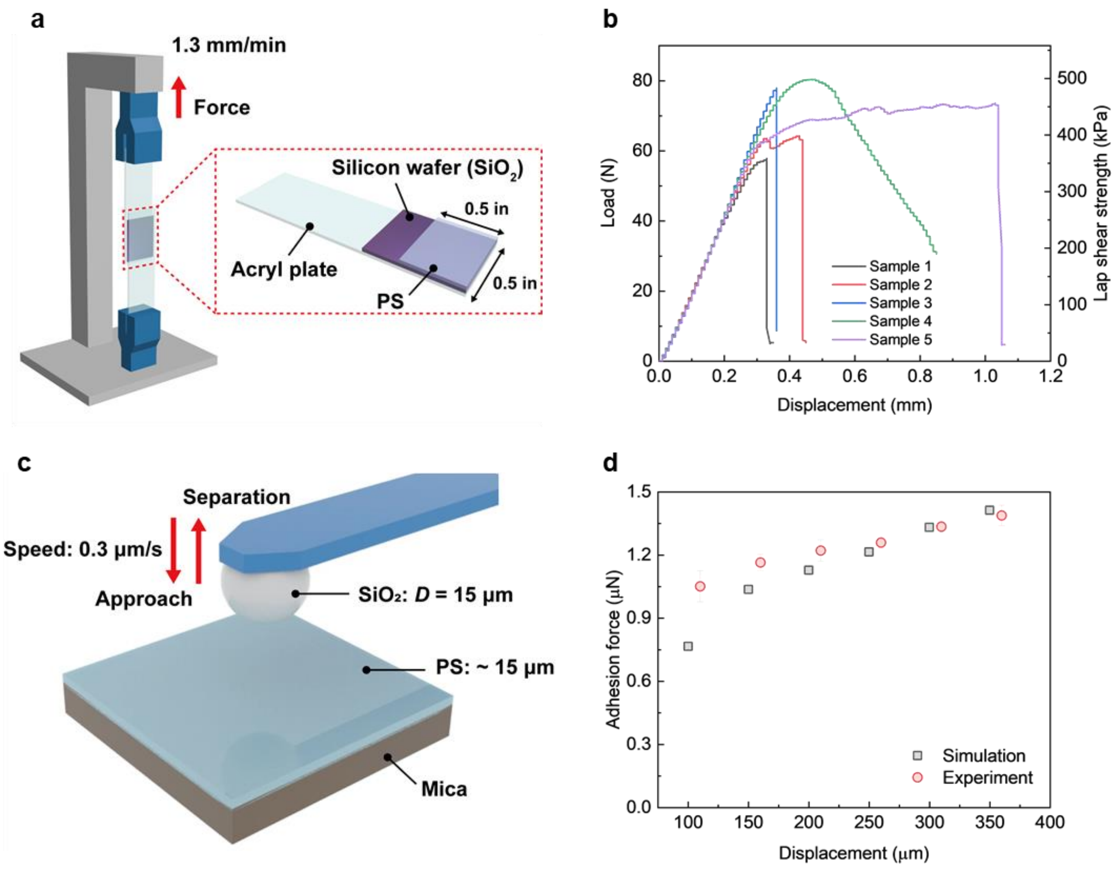

Figure S14. Experiments to determine parameters for the cohesive zone model. a) Schematic illustration of the lap shear test. The sample was composed of acrylic substrate/Si wafer/PS/Si wafer/acrylic substrate. b) Load-displacement curves of five individual samples. The lap shear strength shown on the right Y-axis was calculated by dividing the load on the left Y-axis by the contact area. c) Schematic illustration of the AFM test using the SiO_2 colloidal probe (diameter, 15 μm) on the PS thin film. d) Plots of the adhesion force measured by AFM as a function of the displacement, which was compared to results from the simulation.

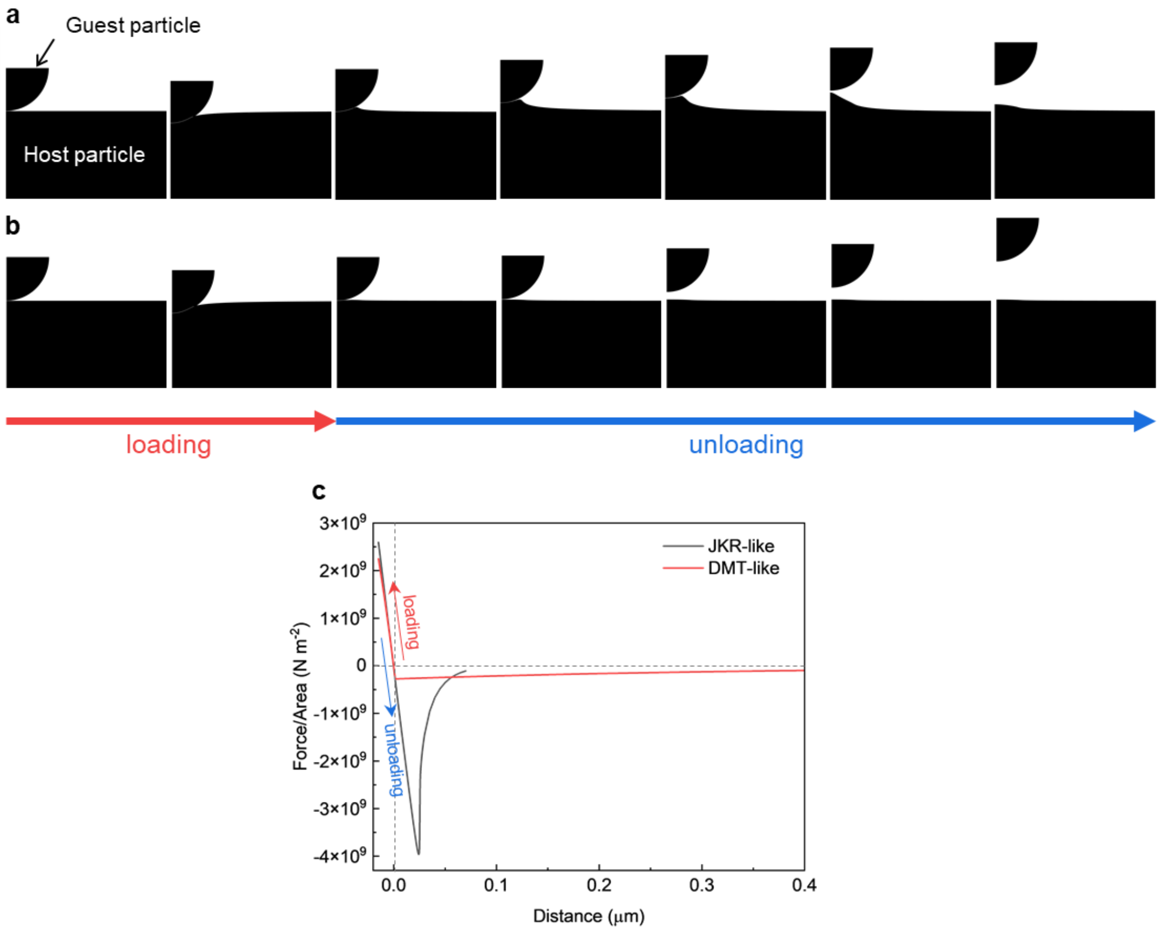

Figure S15. Simulation of the loading and unloading of a guest NP on a host microparticle. Snapshots of (a) JKR-like contact behavior and (b) DMT-like contact behavior. c) Variations in interaction force during contact behaviors.

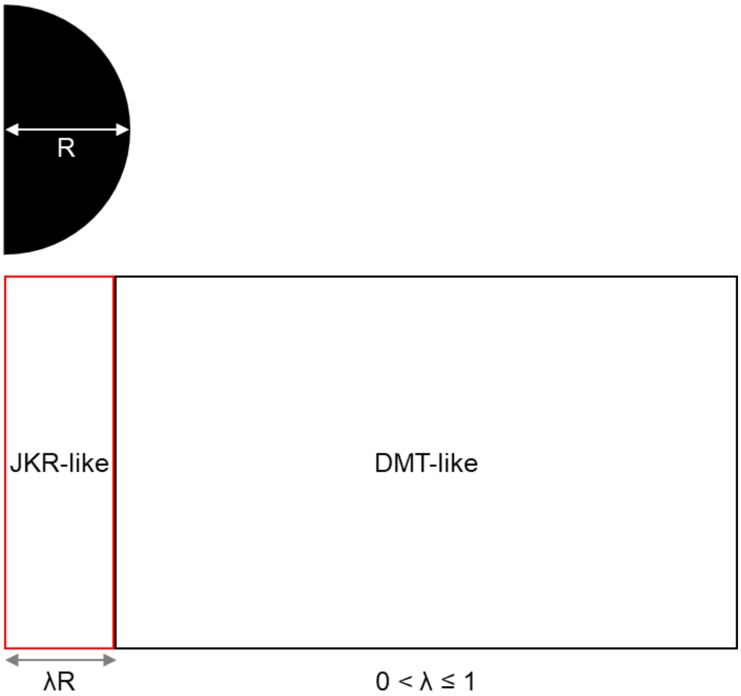

Figure S16. Establishment of regions with different contact conditions for realistic interactions.

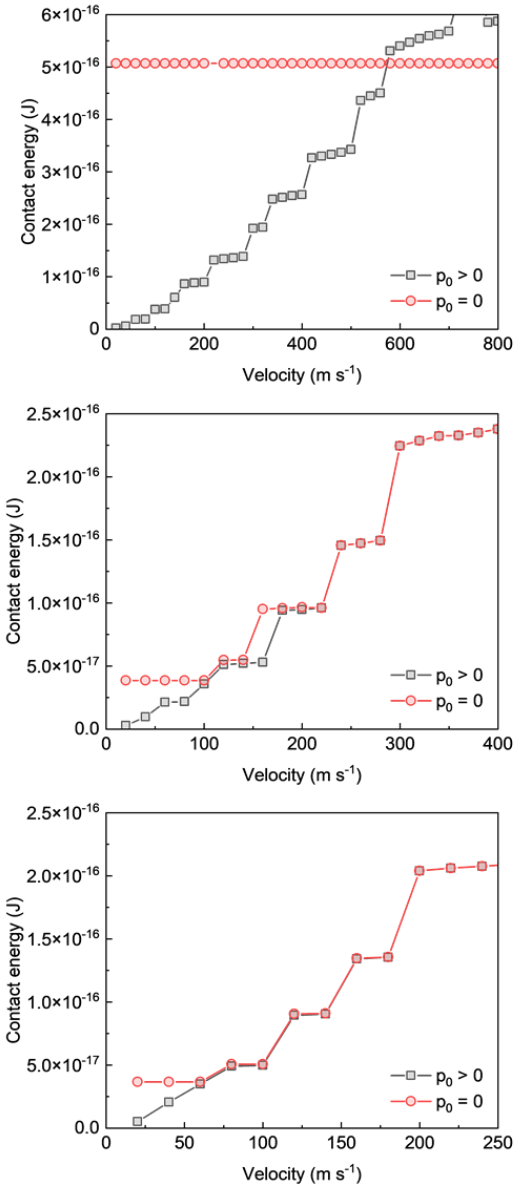

Figure S17. Variations in contact energy with impact velocity under different simulation parameters.

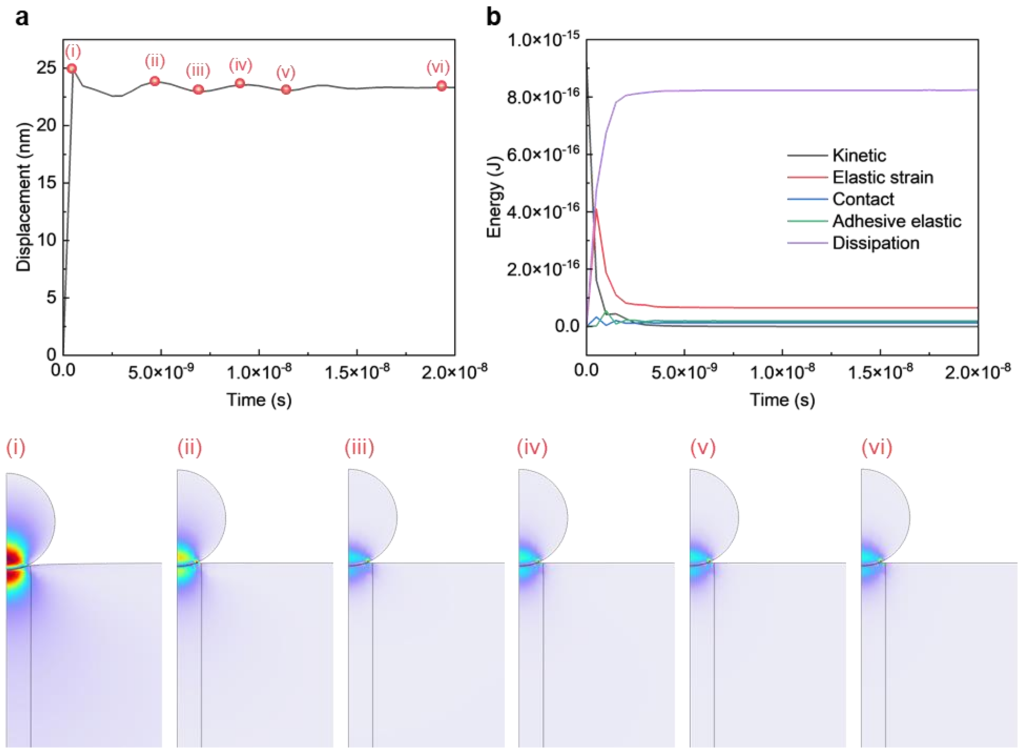

Figure S18. Time-dependent adhesion behavior under an impact velocity of 40 m s^{-1} with simulation parameters, f_p=1.0, p_0=10^{-8} Pa, and λ=0.5. a) Changes in the depth at which a guest particle is embedded in a host particle over time. b) Corresponding changes in energy contribution.

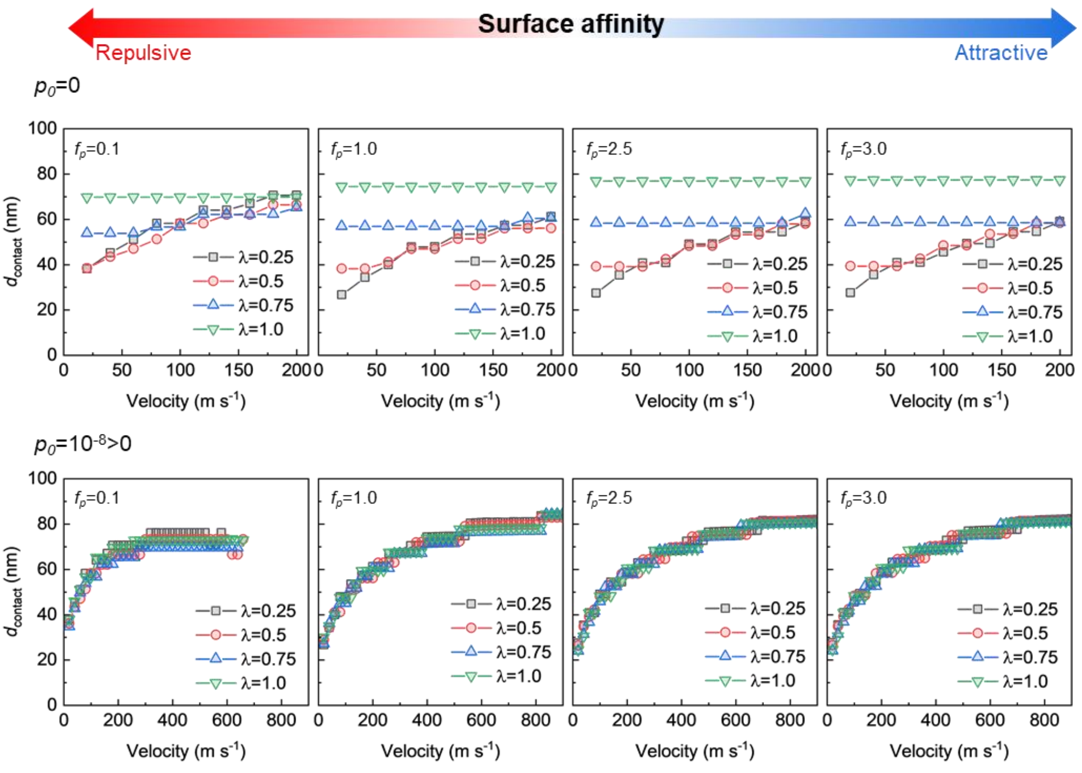

Figure S19. Variation of the contact diameter with impact velocity based on simulation parameters, including f_p, p_0, and λ.

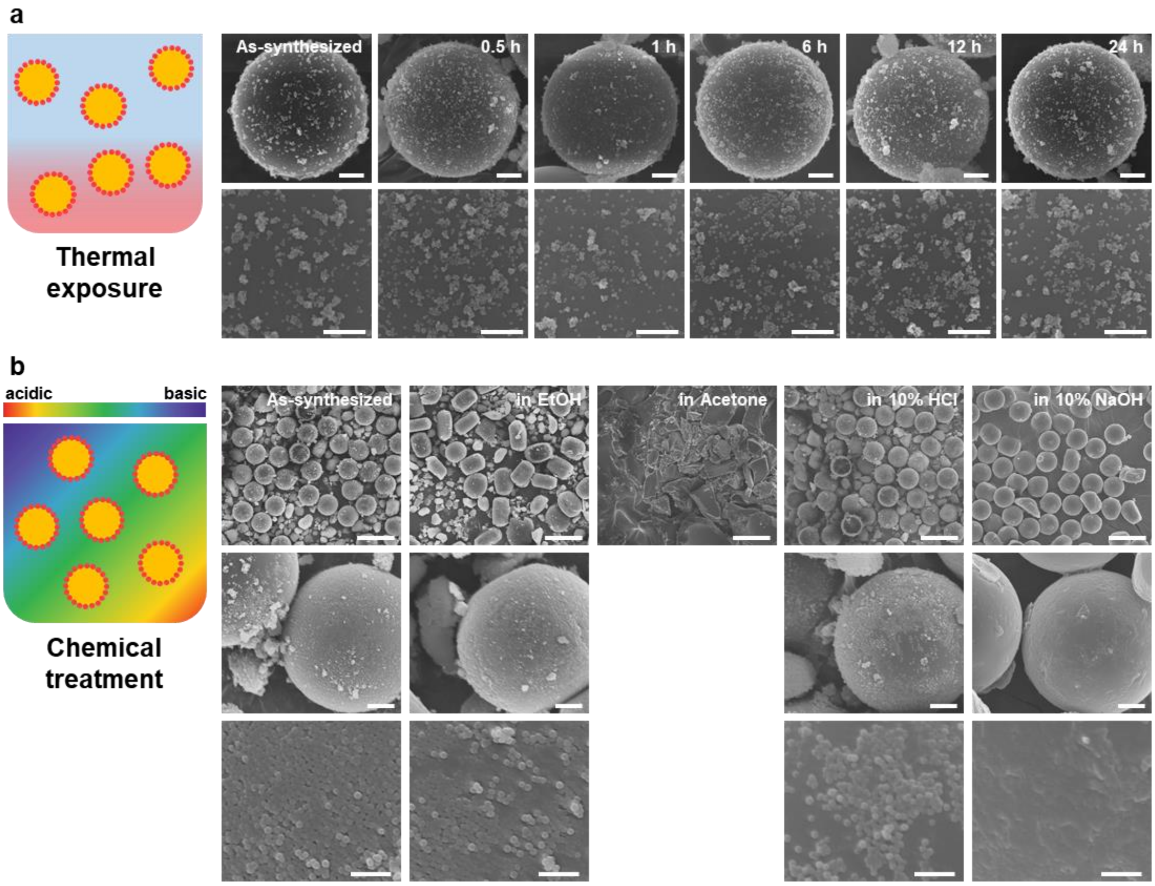

Figure S20. a) SEM images of the SiO_2/PS HSPs after immersion in water at 80 °C for 30 min, 1 h, 6 h, 12 h, and 24 h, respectively, for thermal stability test. b) SEM images of the SiO2/PS HSPs after exposure to various chemical environments including EtOH, acetone, 10% HCl (acidic), and 10% NaOH (basic), compared to the as-synthesized sample for chemical stability test. For (a), the scale bars denote 2 μm for the top row and 1 μm for the bottom row. For (b), the scale bars denote 20 μm for the top row, 2 μm for the middle row, and 500 nm for the bottom row.

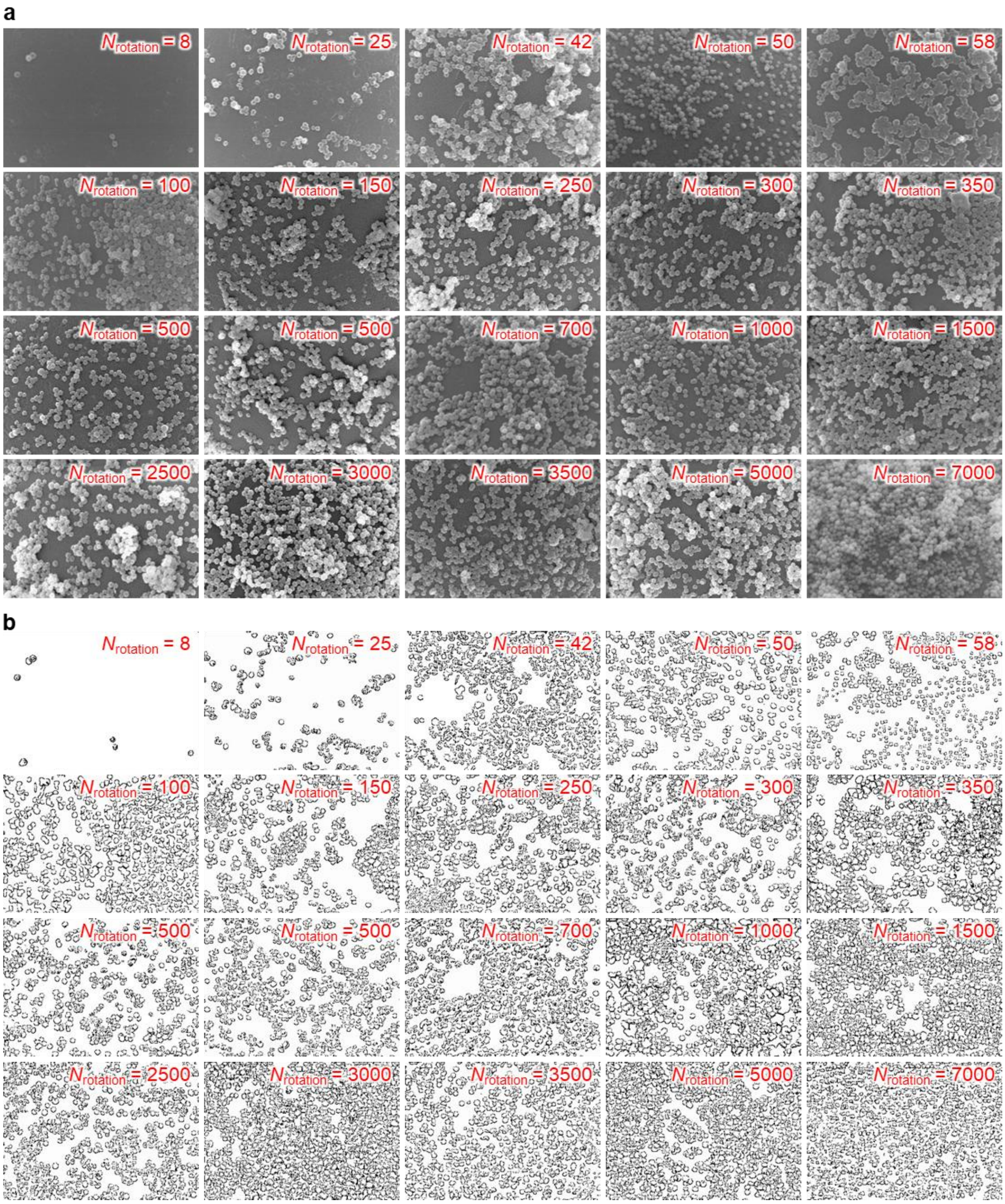

Figure S21. Surface coverage analysis of HSPs. a) SEM images and b) corresponding binarized images of the SiO_2/PS HSPs as a function of $N_{rotation}$. The binarized images were processed using ImageJ software to extract particle distribution. The $N_{particle}$ adhered within a specific region of 3.4 × 4.9 μm^2.

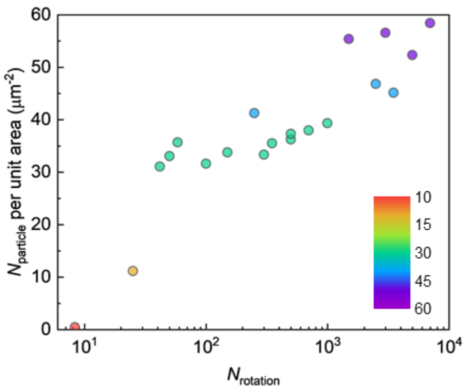

Figure S22. The measured N_{particle} of SiO_2 NPs per unit area from the SiO_2/PS HSPs as a function of N_{rotation}.

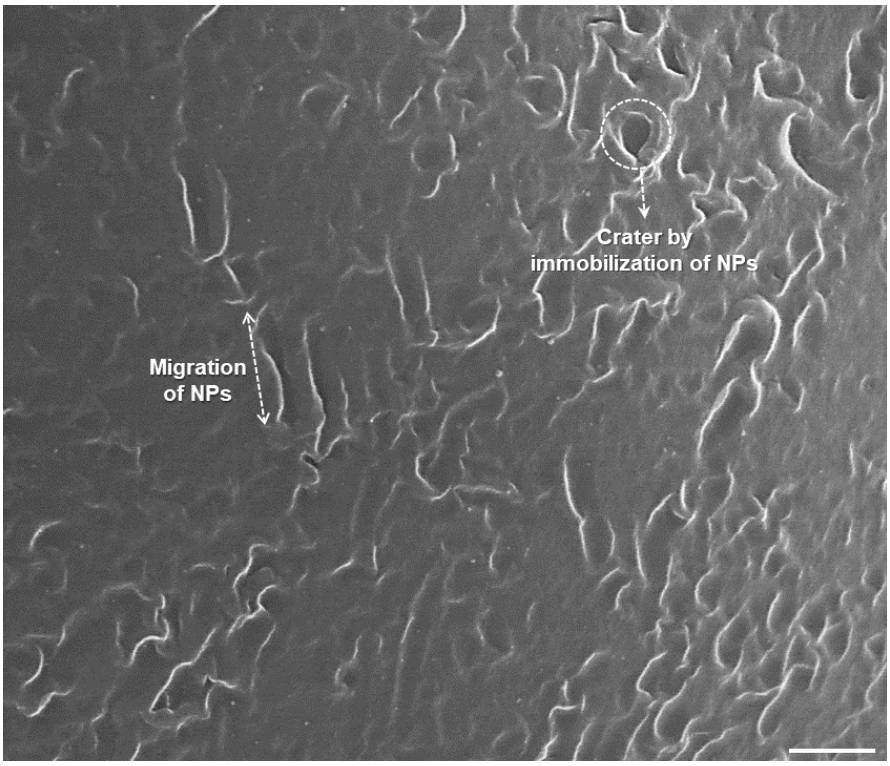

Figure S23. Evidence of the migration of the SiO_2 NPs during collision. SEM image of the SiO_2/PS HSP in which the adhered SiO_2 was removed via etching. The scale bar denotes 200 nm. The HSP was prepared by mixing the PS MPs (diameter, 10 μm) and SiO_2 NPs (diameter, 100 nm) using a magnetic stirrer at 700 rpm for 30 min.

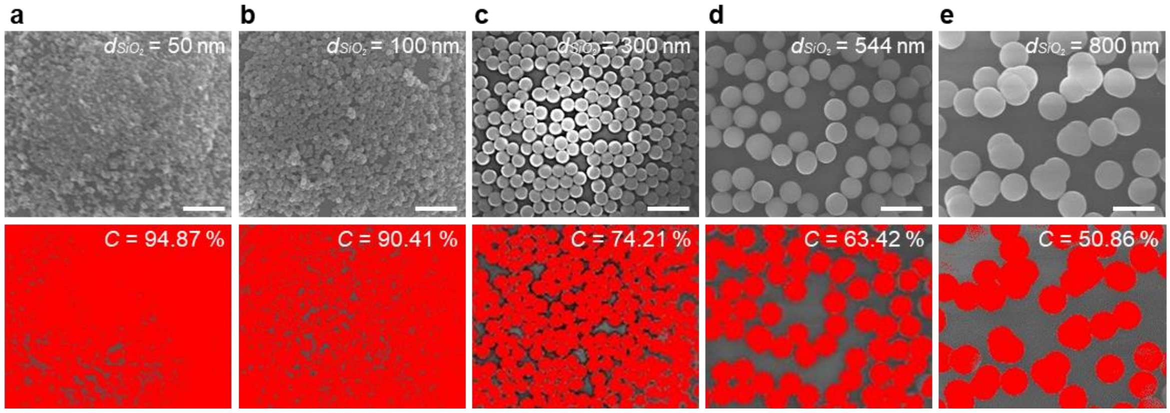

Figure S24. Comparison of surface coverage as a function of the SiO_2 NPs size. SEM images of SiO_2/PS HSPs as a function of diameter of SiO_2 NPs with (a) 50 nm, (b) 100 nm, (c) 300 nm, (d) 500 nm, and (e) 700 nm, respectively. The scale bars denote 1 μm.

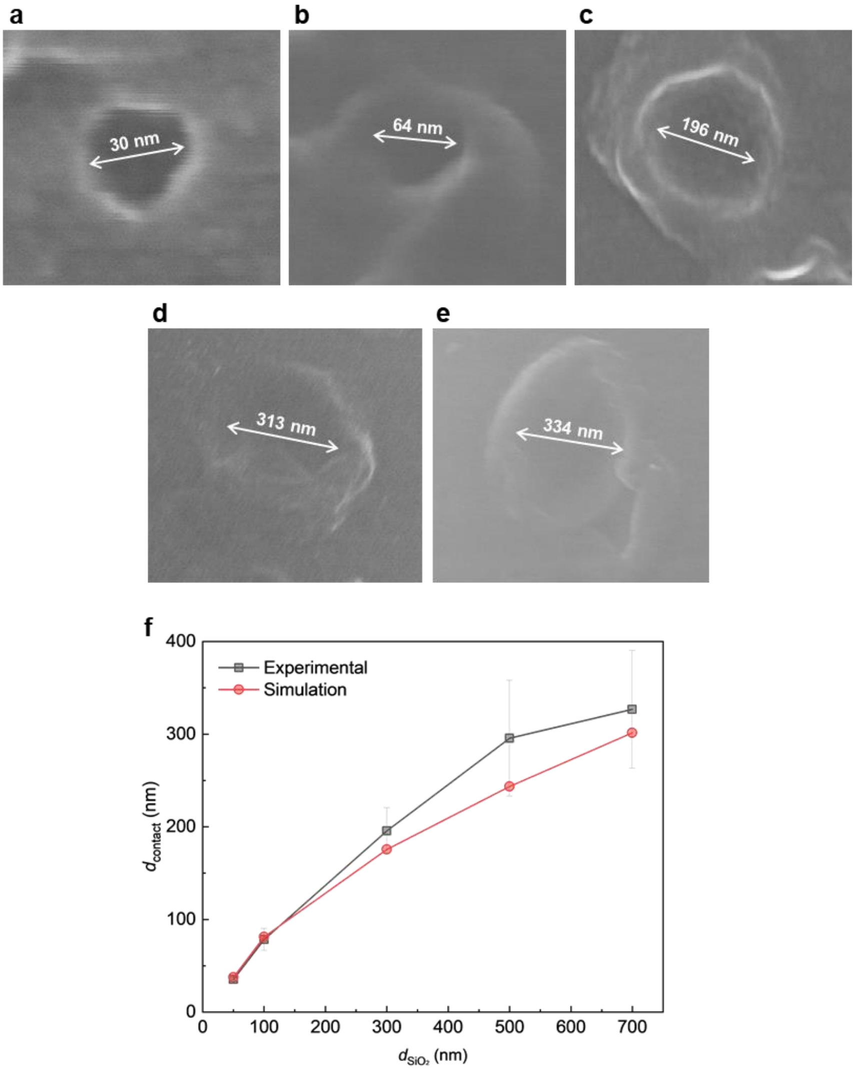

Figure S25. Effect of the size of the SiO_2 NPs on the contact diameter. SEM images of crater shape as a function of a diameter of the SiO_2 NPs with a diameter of (a) 50 nm, (b) 100 nm, (c) 300 nm, (d) 500 nm, and (e) 700 nm, respectively. f) Plots of the contact diameter values as a function of a diameter of the SiO_2 NP.

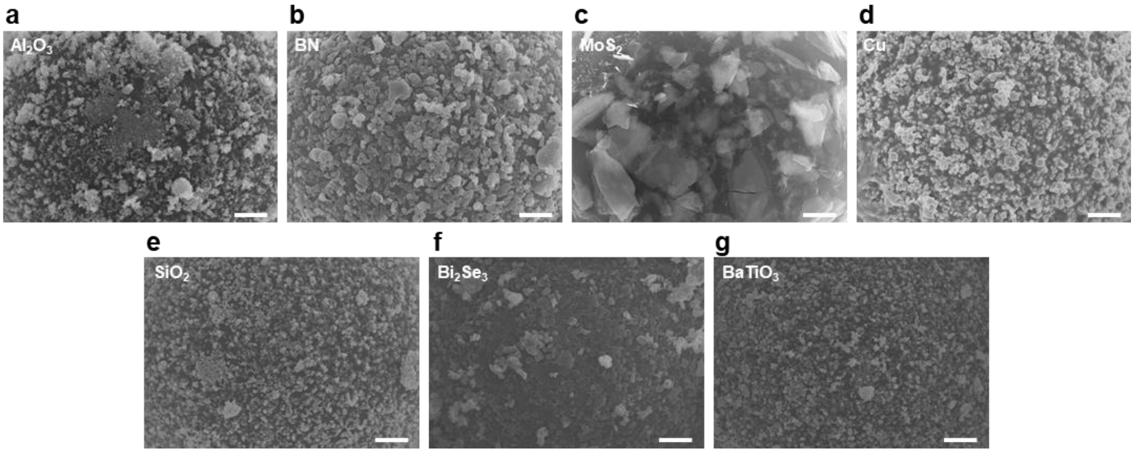

Figure S26. Demonstration of the assembly of various NPs onto PS MPs. SEM images of (a) Al_2O_3, (b) BN, (c) MoS_2, (d) Cu, (e) SiO_2, (f) Bi_2Se_3, and (g) $BaTiO_3$ on PS MPs, respectively. The scale bars denote 1 μm.

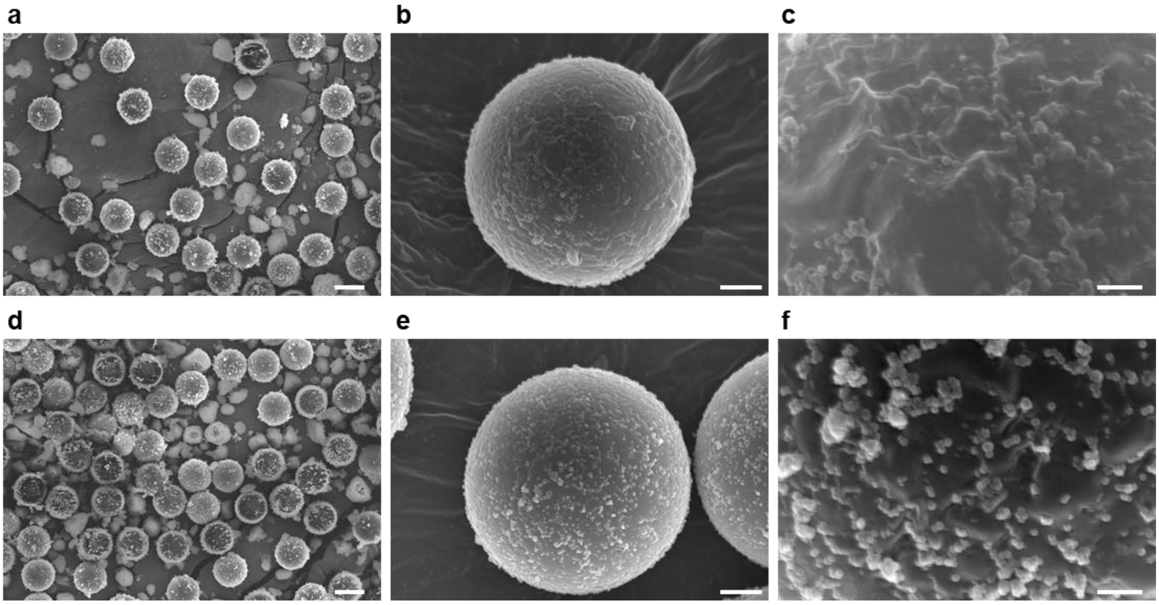

Figure S27. Morphology of the $Cu/SiO_2/PS$ HSPs. SEM images of (a–c) $Cu:SiO_2$ = 1:1 and (d–f) $Cu:SiO_2$ = 1:2 samples, respectively. The scale bars denote 10 μm for (a,d), 2 μm for (b,e), and 500 nm for (c,f).

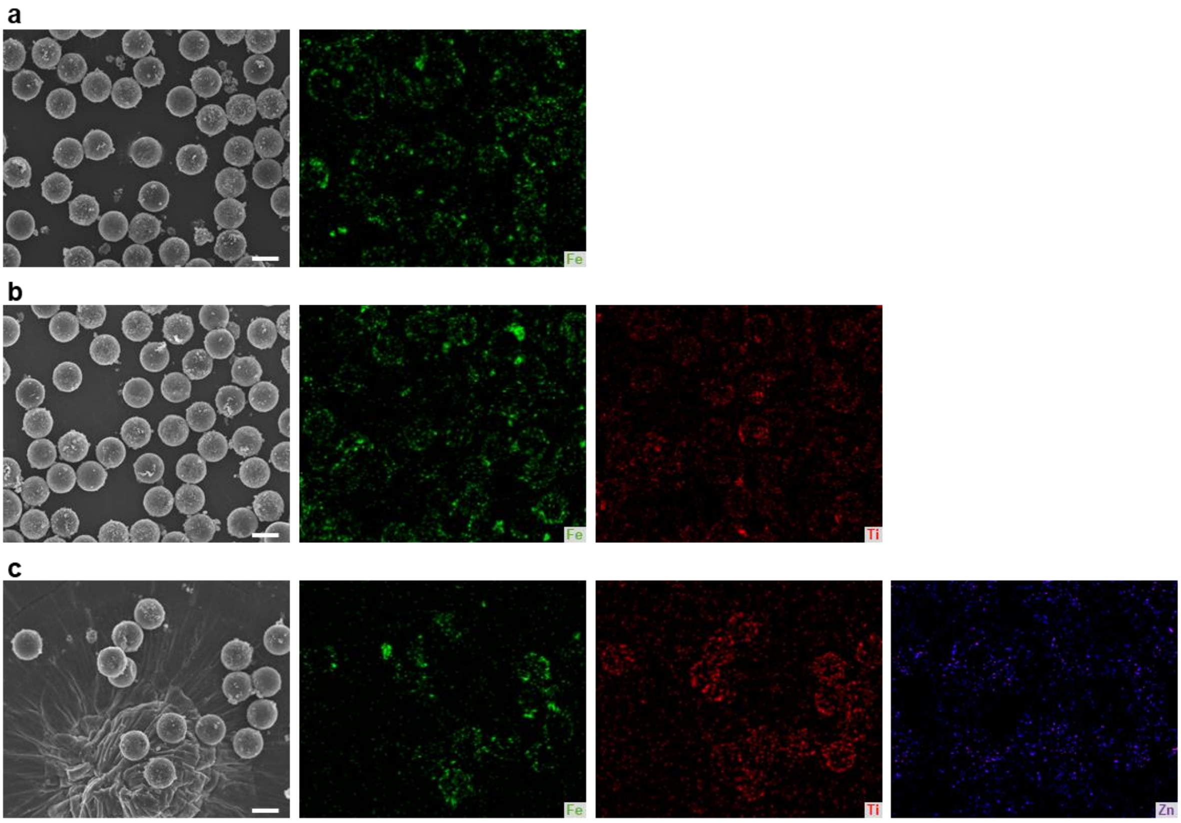

Figure S28. Morphology of the (a) Fe_3O_4/PS HSPs, (b) Fe_3O_4/TiO_2/PS HSPs, and (c) Fe_3O_4/TiO_2/ZIF-8/PS HSPs. In the EDS images, the green, red, and purple colors corresponded to the Fe, Ti, and Zn elements. The scale bars denote 10 μm.

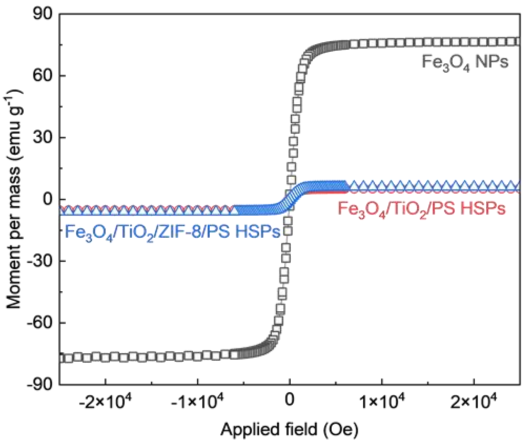

Figure S29. Magnetic responsiveness of the HSPs. VSM magnetization curve of Fe_3O_4 NPs, Fe_3O_4/TiO_2/PS HSPs, and Fe_3O_4/TiO_2/ZIF-8/PS HSPs.

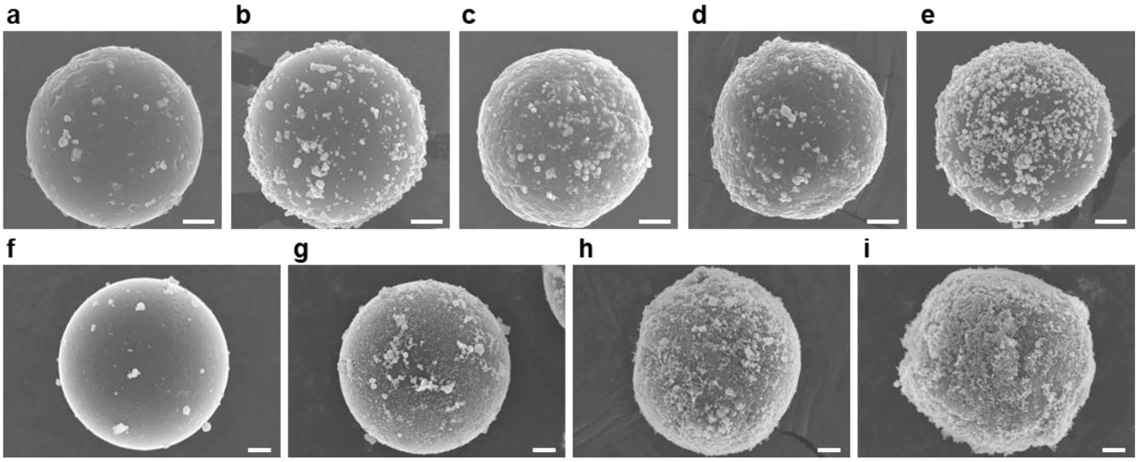

Figure S30. Morphology of the Fe_3O_4/PS and Fe_3O_4/TiO_2/PS HSPs with different compositions. SEM images of (a) 6.7% Fe_3O_4, (b) 7.7% Fe_3O_4, (c) 9.0% Fe_3O_4, (d) 11.1% Fe_3O_4, (e) 20.0% Fe_3O_4, (f) 9.0% Fe_3O_4/0.5% TiO_2, (g) 9.0% Fe_3O_4/0.9% TiO_2, (h) 8.3% Fe_3O_4/8.3% TiO_2, and (i) 6.3% Fe_3O_4/31.3% TiO_2. The scale bars denote 2 μm.

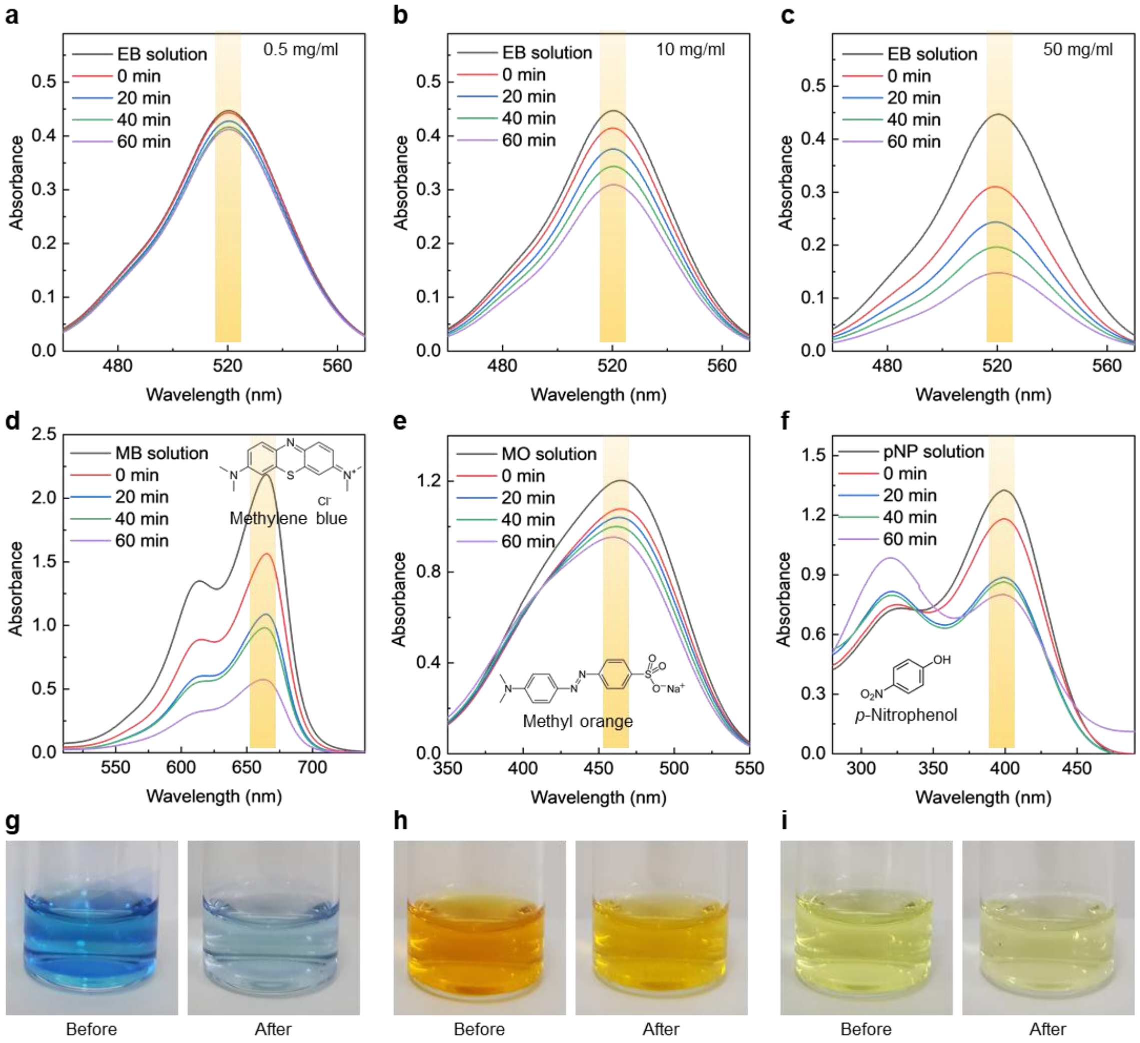

Figure S31. Photocatalytic degradation results of the EB, MB, MO, and *p*NP. a–c) UV-vis spectra of the EB as a function of HSP concentration and time. UV-vis spectra of the (d) MB, (e) MO, and (f) pNP as a function of time. Photographs of (g) MB, (h) MO, and (i) pNP before and after photocatalysis with the HSPs.

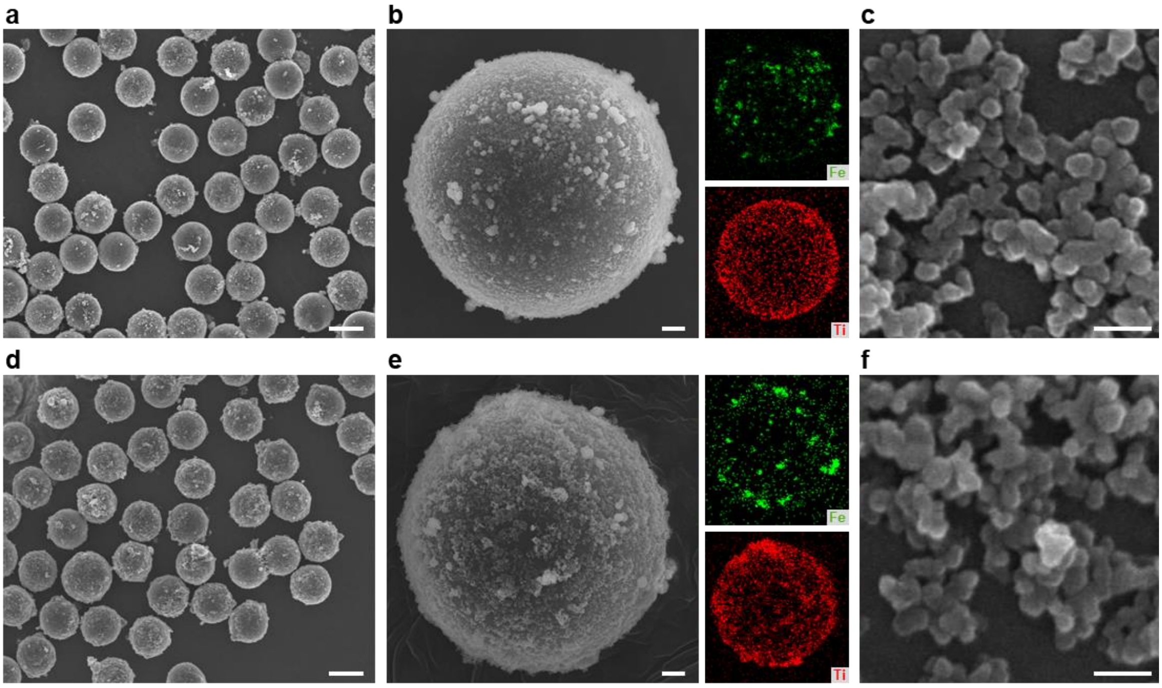

Figure S32. Morphology of the Fe_3O_4/TiO_2/PS HSPs before (a–c) and after (d–f) cycling tests of the photocatalytic degradation. In the EDS images corresponded to the (b) and (e), the green and red colors corresponded to the Fe and Ti elements. The scale bars denote 10 μm for (a,d), 1 μm for (b,e), and 100 nm for (c,f).

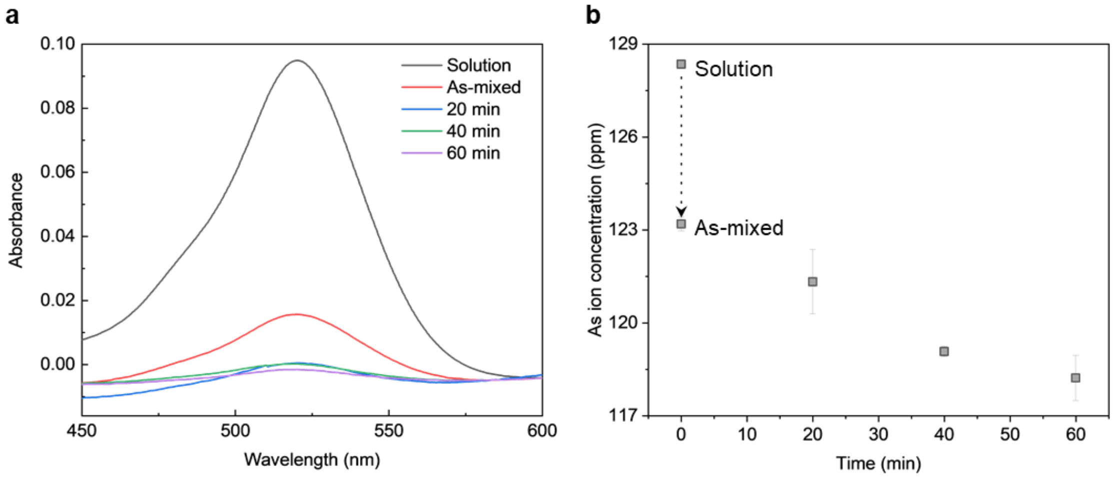

Figure S33. Adsorption behaviors of the As(III) ion with Fe_3O_4/TiO_2/ZIF-8/PS HSPs. a) UV-vis spectrum of the As(III) solution before and after absorption with Fe_3O_4/TiO_2/ZIF-8/PS HSPs as a function of time. b) Plots of the As(III) ion concentration as a function of time.

Table S1. Dispersive and polar components and surface energy values of the Si wafer, APTES-treated Si wafer, and FTMS-treated Si wafer, respectively.

	Dispersive	Polar	Surface energy [mJ m^{-2}]
Si wafer	37.43 ± 0.79	18.02 ± 1.58	55.45 ± 2.36
APTES-treated Si wafer	40.80 ± 0.85	22.15 ± 0.54	62.95 ± 1.40
FTMS-treated Si wafer	25.31 ± 1.00	3.38 ± 1.23	28.69 ± 2.23

Table S2. Key differences between ball milling, sand blasting, and mechanophysical synthesis methods proposed in this study.

	Ball milling	Sand blasting	**Mechanophysical synthesis**
Energy Source	High-energy impact and shear from rotating balls	High-velocity gas-driven abrasive impact	**Low-energy mechanical collision via magnetic stirring**
Process Environment	Enclosed milling chamber; intense mechanical force	Open-air, gas-propelled particles onto rigid substrate	**Enclosed vial, ambient conditions with gentle stirring**
Material Interaction	Bulk powder deformation (milling) or alloying	Surface erosion and roughening of substrate	**Localized deformation of soft polymer (PS), enabling NP embedding**
Deformation Type	Plastic/brittle deformation	Shear-induced abrasion and brittle fracture	**Local plastic deformation at collision sites (no material loss)**
Resulting Structure	Milled powder, phase-transformation, or alloy	Roughened surface	**NPs-immobilized core/shell polymer particles**
Purpose	Reducing powder size or mechano-chemical alloying	Surface modification or texturing	**Formation of hybrid supraparticles (not surface coating or bulk mixing)**

Table S3. Mechanical properties of guest and host particles.

Guest particle (SiO_2)	Elastic modulus	E	70 GPa
	Poisson's ratio	v	0.17
	Density	ρ	2200 kg m^{-3}
Host particle (PS)	Lamé parameter	μ	246.5 MPa
	Bulk modulus	κ	681.25 MPa
	Density	ρ	1 kg m^{-3}
	Bulk viscosity	η_b	0.08 Pa·s
	Shear viscosity	η_v	0.016 Pa·s

Table S4. Parameters for the cohesive zone model.

Tensile strength	σ_t	215.87 MPa
Shear strength	σ_s	36.13 MPa
Tensile energy release rate	G_t	0.0034 N mm^{-1}
Shear energy release rate	G_s	0.0084 N mm^{-1}

Table S5. Quantitative analysis of SiO_2 NPs embedded on the surface of HSPs as a function of $N_{rotation}$.

$N_{rotation}$	$N_{particle}$	θ
8	0.41916	1.82
25	11.13772	12.11
42	31.07784	53.65
50	33.05389	29.80
58	35.68862	58.72
100	31.61677	37.60
150	33.77246	32.50
250	41.25749	54.05
300	33.35329	37.90
350	35.50898	68.95
500	36.22754	40.50
500	37.30539	58.31
700	37.96407	71.92
1000	39.34132	62.70
1500	55.38922	52.60
2500	46.82635	66.29
3000	56.58683	73.00
3500	45.1497	74.35
5000	52.33533	71.90
7000	58.44311	78.70

Table S6. Young's modulus of the particles.

	Young's modulus [GPa]
PS	3.4[14]
SiO_2	74.0[15]
Al_2O_3	392.5[16]
BN	716.3[17]
MoS_2	270.0[18]
Cu	181.0[19]
Bi_2Se_3	50.0~60.0[20]
$BaTiO_3$	67.0[21]

Table S7. Comparison of initially introduced and ICP-OES-determined Cu and Si concentration in the $Cu/SiO_2/PS$ HSPs

	Element	ICP-OES	Initial input
		Average normalized concentration (wt%)	Theoretical concentration (wt%)
$Cu:SiO_2$ 1:1	Cu	63.0	68.1
	Si	37.0	31.9
$Cu:SiO_2$ 1:2	Cu	54.6	51.7
	Si	45.4	48.3

References

[1] M. Achimovičová, F. J. Gotor, C. Real, N. Daneu, *J. Mater. Sci.: Mater. Electron.* **2012**, *23*, 1844.

[2] Y. Pan, K. Sun, S. Liu, X. Cao, K. Wu, W. -C. Cheong, Z. Che, Y. Wang, Y. Li, Y. Liu, D. Wang, Q. Peng, C. Chen, Y. Li, *J. Am. Chem. Soc.* **2018**, *140*, 2610.

[3] D. K. Owens, R. C. Wendt, *J. Appl. Polym. Sci.* **1969**, *13*, 1741.

[4] J. Israelachvili, Intermolecular and surface forces (3rd edition). Academic press, **2011**.

[5] G. Hu, J. Kang, L. W. T. Ng, X. Zhu, R. C. T. Howe, C. G. Jones, M. C. Hersam, T. Hasan, *Chem. Soc. Rev.* **2018**, *47*, 3265.

[6] S. Khan, H. Choi, D. Kim, S. Y. Lee, Q. Zhu, J. Zhang, S. Kim, S. H. Cho, *Chem. Eng. J.* **2020**, *395*, 125092.

[7] J. Huo, L. Xu, J. -C. E. Yang, H. -J. Cui, B. Yuan, M. -L. Fu, *Colloids Surf. A* **2018**, *539*, 59.

[8] A. F. Bower, *Applied Mechanics of Solids*, CRC Press, **2009**.

[9] R. S. Rivlin, *Philos. Trans. R. Soc., A* **1948**, *241*, 379.

[10] B. B. Prasad, F. Duvigneau, D. Juhre, E. Woschke, *Appl. Acoust.* **2022**, *200*, 109059.

[11] K. Mao, M. Y. Wang, Z. Xu, T. Chen, *Powder Technol.* **2004**, *142*, 154.

[12] X. Shi, W. Zhong, Q. Zhao, R. Li, D. Sun, *Sci. Rep.* **2024**, *14*, 9060.

[13] J. C. Simo, T. Laursen, *Comput. Struct.* **1992**, *42*, 97.

[14] G. V. Lubarsky, M. R. Davidson, R. H. Bradley, *Surf. Sci.* **2004**, *558*, 135.

[15] T. Yoshioka, T. Ando, M. Shikida, K. Sato, *Sens. Actuator A Phys.* **2000**, *82*, 291.

[16] S. Ruppi, A. Larsson, A. Flink, *Thin Solid Films* **2008**, *516*, 5959.

[17] S. Zhao, J. Xue, *J. Phys. D Appl. Phys.* **2013**, *46*, 135303.

[18] S. Bertolazzi, J. Brivio, A. Kis, *ACS Nano* **2011**, *5*, 9703.

[19] D. E. J. Armstrong, A. J. Wilkinson, S. G. Roberts, *J. Mater. Res.* **2009**, *24*, 3268.

[20] S. Gautam, A. K. Verma, A. Balapure, B. Singh, R. Ganesan, M. S. Kumar, V. N. Singh, B. Gahtori, S. S. Kushvaha, *J. Electron. Mater.* **2022**, *51*, 2500.

[21] J. Lim, H. Jung, C. Baek, G. -T. Hwang, J. Ryu, D. Yoon, J. Yoo, K. -I. Park, J. H. Kim, *Nano Energy* **2017**, *41*, 337.
